# Cryptic diversity and spatial genetic variation in the coral *Acropora tenuis* and its endosymbionts across the Great Barrier Reef

**DOI:** 10.1111/eva.13435

**Published:** 2022-07-07

**Authors:** Ambrocio Melvin A. Matias, Iva Popovic, Joshua A. Thia, Ira R. Cooke, Gergely Torda, Vimoksalehi Lukoschek, Line K. Bay, Sun W. Kim, Cynthia Riginos

**Affiliations:** ^1^ Institute of Biology University of the Philippines Diliman Quezon City Philippines; ^2^ School of Biological Sciences The University of Queensland St. Lucia Queensland Australia; ^3^ Bio21 Institute, School of BioSciences The University of Melbourne Parkeville Victoria Australia; ^4^ College of Public Health, Medical and Veterinary Sciences James Cook University Townsville Queensland Australia; ^5^ ARC Centre of Excellence for Coral Reef Studies James Cook University Townsville Queensland Australia; ^6^ Gold Coast University Hospital QLD Health Southport Queensland Australia; ^7^ Australian Institute of Marine Science Townsville Queensland Australia

**Keywords:** cryptic species, demographic history, endosymbiont, gene flow, introgression, population genomics

## Abstract

Genomic studies are uncovering extensive cryptic diversity within reef‐building corals, suggesting that evolutionarily and ecologically relevant diversity is highly underestimated in the very organisms that structure coral reefs. Furthermore, endosymbiotic algae within coral host species can confer adaptive responses to environmental stress and may represent additional axes of coral genetic variation that are not constrained by taxonomic divergence of the cnidarian host. Here, we examine genetic variation in a common and widespread, reef‐building coral, *Acropora tenuis*, and its associated endosymbiotic algae along the entire expanse of the Great Barrier Reef (GBR). We use SNPs derived from genome‐wide sequencing to characterize the cnidarian coral host and organelles from zooxanthellate endosymbionts (genus *Cladocopium*). We discover three distinct and sympatric genetic clusters of coral hosts, whose distributions appear associated with latitude and inshore–offshore reef position. Demographic modelling suggests that the divergence history of the three distinct host taxa ranges from 0.5 to 1.5 million years ago, preceding the GBR's formation, and has been characterized by low‐to‐moderate ongoing inter‐taxon gene flow, consistent with occasional hybridization and introgression typifying coral evolution. Despite this differentiation in the cnidarian host, *A. tenuis* taxa share a common symbiont pool, dominated by the genus *Cladocopium* (Clade C). *Cladocopium* plastid diversity is not strongly associated with host identity but varies with reef location relative to shore: inshore colonies contain lower symbiont diversity on average but have greater differences between colonies as compared with symbiont communities from offshore colonies. Spatial genetic patterns of symbiont communities could reflect local selective pressures maintaining coral holobiont differentiation across an inshore–offshore environmental gradient. The strong influence of environment (but not host identity) on symbiont community composition supports the notion that symbiont community composition responds to habitat and may assist in the adaptation of corals to future environmental change.

## 1 INTRODUCTION

Genetic biodiversity is not always packaged into tidy species groups. Rather, dynamic tensions between divergence and homogenization shape genetic diversity among taxa that interbreed occasionally in spatially and (or) temporally variable manners (Duranton et al., 2018). How and when taxa diverge to create reproductive barriers are central problems of speciation (Coyne & Orr, 2004; Roux et al., 2016). But also, how and when distinct taxa solidify or soften their delineations, and whether divergence inevitably results in speciation, remain open questions in evolutionary biology (Galtier, 2019; Hey & Pinho, 2012; Sousa & Hey, 2013). With the increasing use of genomic approaches, it is evident that genomic regions can vary in their permeability to gene flow; regions of the genome that can resist the homogenizing effect of gene flow can maintain local adaptation and facilitate divergence, especially for high dispersal taxa (Andrew & Rieseberg, 2013; Martin et al., 2013; Riquet et al., 2019). Consequently, cryptic diversity can manifest over spatial scales that are expected to be homogenized by gene flow. Characterizing such cryptic diversity and understanding its spatial distribution are important for developing biodiversity management strategies in our rapidly changing world (Hey et al., 2003).

Corals form the basis of reef ecosystems that harbour exceptional biodiversity (Fisher et al., 2015), yet delineation of coral species remains contentious due to frequent mismatches between morphological and molecular systematics, morphological plasticity, cryptic genetic diversity and porous taxonomic boundaries. Genome‐scale investigations provide more robust and better‐resolved estimates of genetic relationships than previous approaches due to the analytic depth afforded by thousands or more loci. Recent genetic studies have revealed surprising divisions and gene exchange among coral taxa, provoking re‐evaluations of both deep evolutionary relationships (Fukami et al., 2008; Kitahara et al., 2014; Ying et al., 2018) and relationships among closely related taxa. For example, population genomic surveys have uncovered distinct genetic clusters within *Acropora* (Cooke et al., 2020; Fifer et al., 2021; Rose et al., 2018; Underwood et al., 2020), *Agaricia* (Bongaerts et al., 2017; Prata et al., 2022), *Montastraea* (Rippe et al., 2021; Sturm et al., 2020), *Pachyseris* (Bongaerts et al., 2021), *Pocillopora* (Smith et al., 2017; van Oppen et al., 2018) and *Siderastrea* (Rippe et al., 2021), where some earlier microsatellite‐based studies had failed to detect these clusters. (For full taxonomic authorities for all species mentioned throughout this paper, see the World Register of Marine Species: www.marinespecies.org). Cryptic differentiation in corals often coincides with environmental gradients, including depth (Bongaerts et al., 2017; Rippe et al., 2021; van Oppen et al., 2018) and inshore–offshore shelf location (Kenkel et al., 2013; Warner et al., 2015), indicating that processes structuring variation can occur on small spatial scales relative to the species' range. There is, thus, an evolutionary tug‐of‐war between processes that drive divergence versus those that maintain genetic cohesion among many closely related coral taxa. Characterizing the breadth and distribution of coral diversity therefore requires sampling across relevant spatial scales and environments and employing analytical approaches that are sufficiently resolute to capture the evolutionary processes contributing to this diversity (as in Bierne et al., 2013).

The cnidarian host's intimate symbiosis with dinoflagellates from the family Symbiodiniaceae further complicates the delineation of corals into discrete species. Symbiont compositions have profound effects on the response of the coral holobiont (or composite organism) to environmental stress (Cooper et al., 2011; Hoadley et al., 2021; Manzello et al., 2019; Morikawa & Palumbi, 2019; Sampayo et al., 2008). Within coral hosts, symbiont compositions can vary spatiotemporally in response to environmental gradients, such as depth (Bongaerts et al., 2010, 2013, 2015) and shelf location (Cooper et al., 2011; Howells et al., 2009; Sawall et al., 2014; Tonk et al., 2013; Ziegler et al., 2015). Inshore habitats generally experience more extreme and variable conditions relative to offshore habitats, including warmer and more variable temperatures, greater turbidity and greater concentrations of inorganic nutrients (Camp et al., 2020; Manzello et al., 2019; Ziegler et al., 2015). Symbionts from inshore corals may also facilitate greater recovery from bleaching relative to those from offshore corals (Manzello et al., 2019). Patterns of symbiont variation across environmental gradients may represent important sources of variation that confer adaptive responses to environmental stress, comprising an important axis of coral genetic variation that is not necessarily constrained by taxonomic boundaries of the cnidarian host. The decoupling of symbionts and host inheritance is likely to typify many broadcast spawning corals, where coral juveniles acquire their endosymbionts from the local environment (Baird et al., 2009; Madin et al., 2016; but see Quigley et al., 2020). Spatial costructuring between symbionts and hosts is sparsely documented, and this study builds on previous studies that simultaneously consider spatial genetic variation of coral hosts and symbionts at the colony level (exceptions include: Bongaerts et al., 2021; Cooke et al., 2020; Kenkel et al., 2013; Rose et al., 2018, 2021; Thomas et al., 2022; van Oppen et al., 2018; Warner et al., 2015).

In this study, we simultaneously examine host and symbiont genetic variation of the reef‐building coral *Acropora tenuis* (Dana, 1846–1849) across the entire GBR, sampling both latitudinal and inshore–offshore gradients. *A. tenuis* is a common branching coral of the Great Barrier Reef (GBR) and the wider tropical Indo‐Pacific, with broadcast spawning reproduction. Previous microsatellite surveys of *A. tenuis* (Lukoschek et al., 2016; Riginos et al., 2019) spanning much of the GBR (1500+ km) found greater genetic diversity in southern GBRs (especially the Swains and Capricorn–Bunker reef groups) compared with central and northern reefs, with subtle signs of possible admixture from the Coral Sea. There was no compelling evidence for cryptic speciation, and symbiont diversity was not examined. In contrast, using low‐coverage whole‐genome sequencing and sampling across ~350 km of inshore central GBR reefs, Cooke et al. (2020) found substantial differentiation of colonies from one site, Magnetic Island, compared with colonies from the other four sampling sites, with low levels of gene flow between Magnetic Island and other locations. In the same study, symbiont mitochondrial haplotypes were differentiated among all five sampling locations. The three locations most affected by riverine plumes, including Magnetic Island, contained symbiont haplotypes not found in the two more maritime locations, consistent with environmental filtering of symbionts. Thus, the initial genomic survey by Cooke et al. (2020) suggests that GBR *A. tenuis* may comprise more than one distinctive gene pool, that is, multiple species sensu Mallet (1995). Moreover, this prior work shows that geographic differentiation of symbionts can exceed that of the host animal.

Here, we characterize *A. tenuis* colonies across the GBR using genome‐wide single nucleotide polymorphisms (SNPs) in the cnidarian hosts and SNPs from symbiont plastids. We aimed to: (1) determine whether *A. tenuis* resolved into two or more cryptic taxa as evidenced by distinct genetic clusters; (2) document spatial extents of such cryptic taxa and their associations with latitude and shelf position; (3) test for gene flow among cryptic taxa using demographic modelling; and (4) characterize patterns of symbiont diversity within and among coral colonies with respect to latitude, shelf position and cryptic cnidarian host taxon identity. Our data suggest that at least three discrete taxa comprise what is currently recognized as *A. tenuis* on the GBR. We demonstrate that diversity in the cnidarian host and its symbionts are costructured by similar environmental processes but with much stronger genetic‐environment associations for symbionts than coral hosts. Additionally, we find that structuring of symbiont variation is independent of genetic identity of cnidarian hosts, suggesting that these discrete but cryptic cnidarian taxa share the same, environmentally determined symbiont pool. This study provides the most spatially comprehensive joint survey of genomic variation in cnidarian hosts and their symbiont partners for any coral species on the GBR to date.

## 2 MATERIALS AND METHODS

### 2.1 Sample collection, DNA extraction and genomic library preparation

**FIGURE 1:**
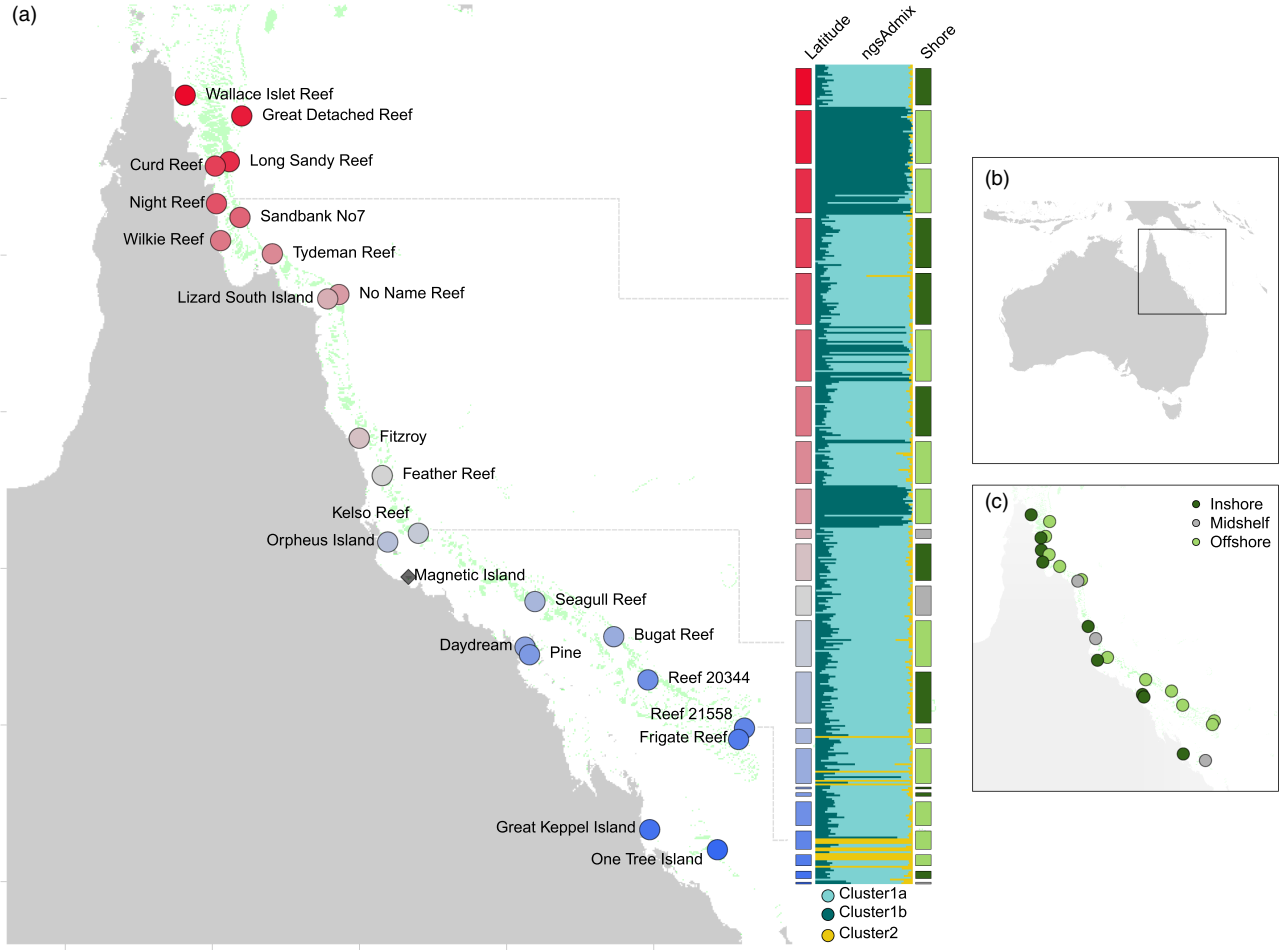
Map of sampling locations across the Great Barrier Reef (GBR) and major genetic clusters of *Acropora tenius* samples based on *NGSadmix*. (a) Geographic locations of *A. tenuis* samples from 23 reefs across the GBR with each location coloured by their latitude. The *NGSadmix*‐derived genetic ancestry proportions and clustering of each of 448 colonies is summarized by the plot (with *K* = 3) at the right side of (a). Bars on the left side of the *NGSadmix* plot indicate sampling locations (coloured as latitude), and bars on the right side show the shore position of the sampling location. Black dashed lines connecting the points on the map to the bars of the *NGSadmix* plot are added for quick reference. (b) Map of Australia illustrating where the sampling locations are situated. (c) Sampling locations coloured based on shore position

Details for all methods including function calls and settings can be found in the Supplemental Information in Appendix S1. Adult colonies from 26 locations along the span of the GBR were sampled (Figure 1 and Table S1). Throughout this paper, we refer to our study organism as *A. tenuis* despite recent evidence that this species on the GBR may be different from the *A. tenuis* type specimen from Fiji and the appropriate GBR species name may be *Acropora kenti* (T. Bridge pers comm; also see comment in Cooke et al., 2020). In the absence of a formal taxonomic revision of the species, we continue with recent conventions to avoid further confusion. Whole genome libraries were prepared and enriched for a selection of ~21,000 nuclear SNPs randomly distributed across the *A. tenuis* genome using custom bait probes (Arbor Biosciences). Libraries for 698 individual colonies and four technical replicates were sequenced on an Illumina HiSeq HT (125 bp paired end reads) to approximately 5× coverage.

### 2.2 Processing, filtering and clone identification from genomic data

*FastQC* (http://www.bioinformatics.bbsrc.ac.uk/projects/fastqc) and *bbtools* (Bushnell, 2014) were used for initial data processing, and *BWA* (Li & Durbin, 2009) was used to map paired reads against the *A. tenuis* draft genome (aten_final_0.11.fasta; Cooke et al., 2020). Data conversion, indexing, sorting and annotations were undertaken with *samtools* v.1.10 (Li et al., 2009) and *picard* (http://broadinstitute.github.io/picard/).

We used *ANGSD* v0.921 (Korneliussen et al., 2014) to identify polymorphic sites and estimate genotype likelihoods in a probabilistic framework that incorporates statistical uncertainty associated with sequencing errors and missing genotypes in low‐coverage genomic datasets (Kim et al., 2011). For low‐coverage genomic data, using genotype likelihoods improves the accuracy of population genetic inferences (Warmuth & Ellegren, 2019) and diversity estimates (Korunes & Samuk, 2021) and *ANGSD* enabled analyses are the most accepted for low‐coverage population genomic data (Matz, 2018; Therkildsen & Palumbi, 2016). For *A. tenuis*, only sites that included 3+ reads in ≥40% of individuals were retained, and only the top 65% of individuals, ranked by the total number of positions covered by 3+ reads passing minimum quality criteria of mapping quality 30 and base quality 30, were kept.

Potential clones were identified following the approach of Manzello et al. (2019), where this method identifies likely clones by applying a threshold on genetic similarities estimated as identity‐by‐state (IBS) between individuals. Four pairs of technical sequence replicates allowed us to determine the upper 95% confidence interval for pairwise genetic differences (i.e. 1 − IBS) for multilocus genotypes (MLG). We applied this upper limit as a threshold and then pruned sets of individuals falling below this value to a single representative.

### 2.3 Identification of major host clusters, verification of taxonomy and integration with previous *A. tenuis* studies

Genotype likelihoods were used to infer genetic clusters among our samples with *ANGSD*. The sites used for this analysis required minimum criteria of mapping quality 30, base quality 30 and coverage ≥3 reads in 85% of individuals. We called major and minor alleles directly from the genotype likelihoods assuming biallelic sites with a likelihood ratio test *p*‐value <0.000001. An individual covariance matrix was extracted using *PCAngsd* (Meisner & Albrechtsen, 2018) with a 0.05 minor allele frequency (MAF) threshold, and the optimal number of ancestral populations (K genetic clusters) was determined using the—‘‐admix’ option. Principal components analysis (PCA) was performed using R (v4.0.1; R Core Team, 2019). The Bayesian hierarchical clustering method *NGSadmix* (Skotte et al., 2013) was applied to estimate individuals' ancestry proportions assuming 2–6 K genetic clusters (MAF = 0.05). Our data were most consistent with three distinct genetic clusters, also inferred by *PCAngsd*, which we refer to as Clusters 1A, 1B and 2 (see Section 3).

To contextualize the taxonomic relationships of these clusters with other *Acropora* species, we identified a subset of samples that were representative of their genetic clusters (assignment probability *q* ≥ 0.85 obtained through *NGSadmix*, see Supplemental Information in Appendix S1 for more details). We performed an analysis of ultraconserved element (UCE) and exon consensus sequences to confirm that specimens used in this study were closely related to the specimens used to build the *A. tenuis* reference genome and the samples from Cooke et al. (2020). UCEs and exon loci have broad utility for resolving evolutionary relationships within the Anthozoa (Quattrini et al., 2018) and species‐level relationships within the Acroporidae (Cowman et al., 2020). We extracted a subset of our representative samples with the greatest number of mapped reads (Cluster 1A, *n* = 14; Cluster 1B, *n* = 14; and Cluster 2, *n* = 6) and combined them with two previously published samples (from Magnetic Island and Fitzroy Island: Cooke et al., 2020), and aligned them to loci extracted from the *A. tenuis* reference genome and to two outgroup species (*Acropora millepora* and *Acropora digitifera*). To extract UCE and exon sequences from whole‐genome data (from Cooke et al., 2020), we performed an in‐silico target‐capture and locus assembly process (detailed in the Supplemental Information in Appendix S1). We then performed maximum likelihood phylogenetic inference based on these alignments using IQ‐Tree (ver. 2.1.2: Minh et al., 2020).

Complementing the UCE/exon phylogenetic analyses, we also undertook phylogenetic analysis using *A. tenuis ITS1* sequences. Using all representative coral samples (as above), we extracted read alignments at the *ITS1* region and obtained consensus sequences using *bcftools* v1.3 (Danecek et al., 2021). A neighbour‐joining tree of *Acropora ITS1* sequences was constructed by combining our *ITS1* consensus sequences with reported *Acropora ITS1* sequences available in GenBank. Alignment (msa v1.20.1 package: Bodenhofer et al., 2015), DNA substitution model evaluation (phangorn v2.6.2 package: Schliep, 2011), genetic distance and neighbour‐joining tree construction (ape v5.4‐1 package: Paradis et al., 2004), and tree visualization (ggtree package: Yu et al., 2017) were undertaken in R.

Additionally, to better understand spatial distributions of major genetic clusters, we visualized our data together with previously published *A. tenuis* populations from five inshore reefs (*n* = 148: Cooke et al., 2020) using genotype likelihoods as inferred by *ANGSD* and PCA in *PCAngsd*. Despite exploring a variety of filtering strategies, the combined dataset showed slight but consistent evidence of a study (or batch) effect (see Section 3 for details), and no further analyses were undertaken that included data from Cooke et al. (2020). We also performed a clustering analysis in *STRUCTURE* (Pritchard et al., 2000) for a set of overlapping colonies (Table S1) from this study and genotyped by Lukoschek et al. (2016) with microsatellites using the same *STRUCTURE* run conditions as the original study.

### 2.4 Population genetic structure and demographic analysis among host genetic clusters

To evaluate genetic structure within clusters and demographic histories between clusters, we focussed on individuals with *q* ≥ 0.85 from *NGSadmix* (Cluster 1A, *n =* 141, Cluster 1B, *n =* 92 and Cluster 2, *n =* 14), as these individuals exhibit low admixture and therefore are most characteristic of their genetic cluster (Figure 1). We calculated pairwise *F*_ST_ between genomic clusters and between reefs within clusters, considering only reefs with at least three sampled individuals. We generated unfolded 2D‐SFS for each comparison and calculated pairwise *F*_ST_ for each site and in overlapping 50 kb windows (10 kb step size). We calculated pairwise geographic distances from latitude and longitude using the Vicinity formula for ellipsoids that accounts for the Earth's curvature and tested for isolation by distance (IBD) between reefs using Mantel tests with 1000 permutations in the R package *vegan* v2.5‐7 (Oksanen et al., 2017).

We performed model‐based simulations in *dadi* v1.7.0 (Gutenkunst et al., 2009) to examine population demographic history, and to test for the presence of gene flow between major clusters, by evaluating the likelihood of different demographic models to the joint site frequency spectra (SFS) between clusters. Although discrimination between ancestral and derived alleles may lend greater inferential power to SFS‐based methods, unfolding SFS relies on accurate assignment of ancestral and derived allele states. However, deep evolutionary divergences between *A. tenuis* and available outgroup genomes (e.g. 15 million years between *A. tenuis and A. digitifera*; Mao et al., 2018 and among the most divergent but undated branches within *Acropora* for *A. tenuis – A. hyacinthus*; Cowman et al., 2020) preclude our ability to infer accurate ancestral states: outgroup sequences are likely to represent independently fixed, lineage‐specific mutations rather than ancestral alleles or may lack orthologous sequences. We therefore performed all *dadi* analyses using folded SFS to avoid the risk of incorrect ancestral assignments and false demographic inferences. Cooke et al. (2020) also used unfolded SFS for *A. tenuis* whole‐genome sequences. We used *ANGSD* and the ‘realSFS’ method to calculate cluster‐specific SFS using the quality criteria as stated above.

We used *dadi* to examine four pairwise demographic models: (i) a divergence model (div), which represents strict isolation with no gene flow between clusters; two isolation‐with‐migration (IM) models with (ii) symmetric and (iii) asymmetric gene flow; and (iv) a secondary contact (SC) model, which entails strict isolation followed by SC with gene flow. To balance sample sizes and improve computational efficiency, we subsampled individuals from clusters 1A (*n* = 15) and 1B (*n* = 13) to match the low total number of individuals in Cluster 2 (*n* = 14) and prioritized individuals with the highest depth of coverage. From Cluster 1A, we used three groups of individuals (each group composed of 15 samples) sampled in the northern (Wilkie Reef, *n* = 9; Sandbank Reef, *n* = 6), central (Fitzroy Reef, *n* = 7; Kelso Reef, *n* = 8) and southern (Bugatti Reef, *n* = 6; Daydream, *n* = 1; Pine, *n* = 2; Reef 20‐344, *n* = 4; Seagull Reef, *n* = 2) GBR to represent the large spatial coverage due to detected IBD within this cluster. For each cluster and each group, we selected individuals that were from the same or nearby reef(s). Using these subsets of individuals, we then generated seven different joint‐SFS representing seven comparisons between three Cluster 1A datasets (north, central and south) and Cluster 1B (north) and Cluster 2 (south). Our pairwise comparisons were thus made between sympatric corals (northern Cluster 1A with Cluster 1B; and southern Cluster 1A with Cluster 2), and allopatric corals (central and southern Cluster 1A with Cluster 1B; northern and central Cluster 1A with Cluster 2; and Cluster 1B with Cluster 2). All the folded joint‐SFS were generated using *ANGSD* v0.934 following the same settings as described for single population SFS. For each of the seven joint‐SFS, we estimated the likelihood of our four different models and estimated their parameters following the optimization of Portik et al. (2017; see Supplemental Information in Appendix S1). Divergence dating followed the same assumptions as detailed by Cooke et al. (2020).

### 2.5 Spatial distribution of cnidarian host genetic clusters relative to latitude and shelf position

To characterize how genomic variation in coral hosts was distributed across the GBR, we performed a partial redundancy analysis (RDA) to partition the effects of latitude and shelf position on coral host genotypes. We fit the model:

$$Y=\text{Latitude}+\text{Shore}\;|\;\left(\mathrm{PC}1+\mathrm{PC}2\right).$$

**FIGURE 2:**
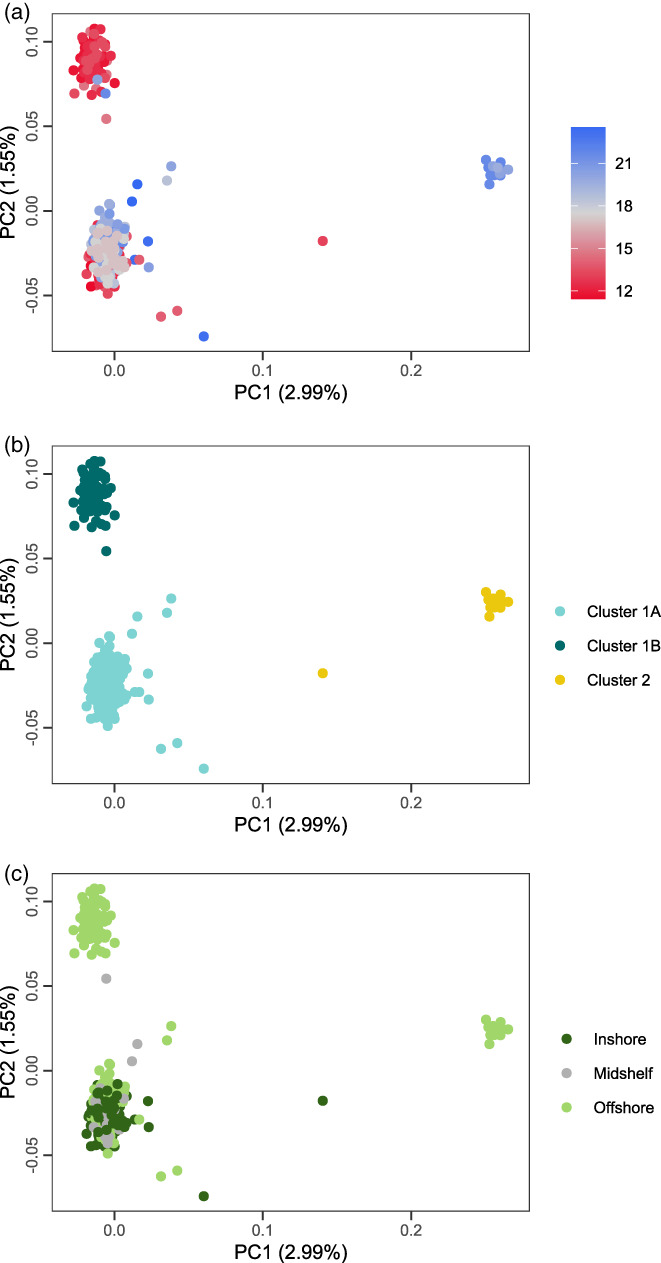
Population genomic structure in *Acropora tenuis*. Scatterplots illustrate the first two principal component axes separating *A. tenuis* samples into three major genetic clusters. (a) Samples coloured by latitude south. (b) Samples coloured by genetic cluster, where *q* > 0.50 was used to determine cluster assignment. (c) Samples coloured by shore position. See respective legends for colour keys

Here, our response, **Y**, was a matrix of scaled inferred individual allele frequencies ranging from 0 to 1: 0, a homozygote for the major allele; 0.5, a heterozygote; and 1, a homozygote for the minor allele. **Y** comprised 448 coral samples and 14,789 SNP loci. The response matrix was conditioned on PC scores of genomic variation (that is, PC1 and PC2; Figure 2) before testing the effects of our focal constraining predictors. The effect of latitude was fit as a continuous predictor (scaled to a mean of 0 and variance of 1), and the effect of shore was fit as a categorical predictor (inshore, midshelf and offshore). The inshore factor was set as the reference factor level for the shore effect. RDA was executed using *vegan* package in R (Oksanen et al., 2017).

### 2.6 Assaying symbiont diversity using ITS2 and plastid genomes

Our data on symbionts include only short reads obtained from shotgun sequencing of the coral colony, and therefore, typical approaches based on matching longer sequence reads to reference databases were not feasible. Rather, we draw upon strategies for examining allelic variation in pooled sequence data and focus on the community of symbionts within each coral as a pooled sample. We began analyses of symbiont variation by testing whether there were compositional differences of *ITS2* symbiont clades among coral host genetic clusters. A reference set of Symbiodiniaceae *ITS2* sequences was obtained from the SymPortal database (Hume et al., 2019); we used the ‘published named’ sequence set. We mapped short reads of all coral samples to these symbiont *ITS2* sequences using *bwa mem* and alignments were quality filtered with *samtools* v1.3 for a MAPQ ≥20. A custom R script was used to parse SAM files to obtain haplotype matches. A minimum of 3 mapped reads was required as evidence of a symbiont *ITS2* reference in a coral sample. We only observed hits to *ITS2* reference sequences from species of the genus *Cladocopium* with little variation in *ITS2* among colonies (see Section 3). Note that we did not attempt to define intragenomic variants as described by Hume et al. (2019); rather, we used the SymPortal reference only to verify that our *ITS2* samples matched *Cladocopium*. Then, we proceeded with downstream analyses from the perspective of within‐genus symbiont variation and focussed on sequence diversity in plastid genome of *Cladocopium goreaui* (LaJeunesse et al., 2018), where within‐colony polymorphism should represent orthologous diversity among strains (without additional paralogous diversity within strains as typified by *ITS2*).

Our novel approach of using plastid genome allele frequencies to characterize symbiont variation is analogous to that well established in pool‐seq studies, whereby the goal is to estimate the composition of allele frequencies in a pooled sample and not determine individual genotypes or haplotypes (Schlötterer et al., 2014). Thus, we describe the ‘population’ diversity of symbionts for each coral colony. Illumina short reads were mapped to the *Cladocopium* plastid genome (Clade C1: Liu et al., 2018) to identify SNP and indel variants, herein referred to as ‘symbiont plastid loci’. Details of our custom mapping and filtering procedures are detailed in the Supplementary Methods in Appendix S1. Our final data set of symbiont allele frequencies comprised *n* = 223 coral samples, with no missing data. The median number of alleles per locus was 2, with a maximum of 17 alleles, and a 75th and 97.5th percentile of 3 and 15.65 alleles. As is the norm with pooled short‐read data, we are unable to phase alleles, but can use allele counts (frequencies) to (i) measure symbiont community diversity within each coral colony, and (ii) characterize the distribution of symbiont diversity among different coral taxa or across environmental gradients.

### 2.7 Spatial distribution of symbiont plastid allele diversity relative to latitude and shelf position

To test how symbiont allelic variation was distributed among coral hosts, we performed two analyses: a generalized linear model (GLM) for the number of symbiont alleles and an RDA. Our GLM of the number of symbiont alleles was used to assess how symbiont diversity varied over latitude, shelf position and coral host. We determined the number of unique alleles per plastid locus for each sampled coral colony:

$$N_{A}=\text{Counts}+\text{Latitude}+\text{Shore}+\text{Host cluster}+\text{Locus}+\text{Sample}.$$

Here, *N*_A_ was the number of alleles at a locus, counts were the number of mapped reads at a locus, latitude and shore reflect colony position, host cluster was based on PCAs of the host, and locus and sample were fit as random effects to allow a unique intercept for each plastid locus and each coral colony, respectively. All variables were scaled and normalized. The GLM was fit using the *lme4* v1.1‐27.1 R package (Bates et al., 2014).

A partial RDA was used to examine symbiont structure among corals and across space. In this analysis, we were interested in the allelic composition of symbionts, that is, which alleles were present and their relative abundance in a coral host.

Y=Latitude+Shore+Host cluster∣Counts.

Here, **Y** was the symbiont allele count matrix, and the remaining variables were as above for the GLM. Both the GLM and the partial RDA were conditioned on read counts to control for the effects of greater sequencing effort at a plastid locus.

## 3 RESULTS

### 3.1 Processing, filtering and clone identification from genomic data

We sequenced a total of 702 samples including four sequencing replicates. These samples yielded on average 4,088,885 reads (SD = 3,060,236 reads). After the initial filtering, each sample retained a mean of 3,181,264 reads (SD = 22,850,422). Alignment of the filtered sequences to the reference genome resulted in an average 94% mapping rate (SD = 11%) with ~5% of samples having a mapping rate <73%. The baited regions (including the 500 bp regions flanking them, covering a total of 2,172,865 positions) had a mean coverage of 2.01 reads (across all the 702 samples; SD = 2.89), with read depth ≥3 for 25% of SNPs. The final high‐quality data set resulted in 463 individuals and 296,667 loci falling within baited and nonbaited genomic regions (Table S1), where minimum inclusion criteria are detailed in Table S2. Many of the samples were collected in 2010 or earlier and not specifically stored for genomic work; thus, a high rate of individual dropout was expected and was consistent with visual inspection of total DNA visualized on agarose gels indicating substantial DNA degradation for many specimens.

We classified putative clones as individuals showing levels of pairwise genetic distances (1‐IBS) below a threshold of 0.17, the upper 95% confidence limit among technical replicates. We identified 11 putative clones as evidenced by shared MLG from eight reefs based on these criteria: Tydeman Reef (*n* = 1), Fitzroy Island (*n* = 2), Kelso Reef (*n* = 2), Orpheus Island (*n* = 1), Seagull Reef (*n* = 1), Bugatti Reef (*n* = 2), Pine Island (*n* = 1) and Reef 20‐344 (*n* = 1). After filtering, reducing MLGs to a single representative colony, and removing technical replicates, we retained 60,733 high‐confidence loci falling within baited and nonbaited genomic regions (Table S1) across 448 individuals from 23 reefs (Figure 1).

### 3.2 Identification of major host clusters, verification of taxonomy and integration with previous *A. tenuis* studies

Principal components analysis of 14,789 loci (MAF ≥ 0.05) in *PCAngsd* revealed three discrete genetic groups (Figure 2) with 1.55% and 2.99% of genetic variation explained on the first and second PCs. We observed strong structuring of coral groups by both latitude and inshore–offshore reef position. PC1 separated two larger genetic groups (Cluster 1A and Cluster 1B) from a small group of individuals (Cluster 2) sampled in the southern GBR (Bugatti Reef, Reef 21‐558, Seagull Reef, Frigate Reef). Cluster 1A and 1B were separated on the second PC. Cluster 1A was geographically widespread and represented by individuals sampled from all reefs, except for Great Detached Reef. In contrast, Cluster 1B consisted entirely of individuals sampled from offshore reefs, with almost all individuals originating from northern reefs below 14.7 latitude; two individuals belonging to Cluster 1B are from the southern GBR (Bugatti Reef and Reef 21‐558), suggesting that this taxon is not restricted to the northern GBR, but likely widespread and sympatric with Cluster 1A. Cluster 2 was restricted to southern offshore reefs, although the group had the fewest samples. *NGSadmix* analyses showed concordant groupings for *K* = 3 clusters, with nine individuals showing mixed admixture proportions >0.30 (Figure 1). Notably, one individual showed mixed ancestry from divergent Clusters 1A and 2 (Night Reef) and showed an intermediate position on the PCA (Figure 2).

Extracted UCE and exon consensus sequences for phylogenetic analyses yielded 1681 loci with 207,000 variable sites across all samples. A maximum likelihood tree built using IQ‐Tree clearly resolved the outgroup species and placed all the samples from this study in a single well resolved (1.0 node support) clade containing the *A. tenuis* reference sequence (Figure S4).

After filtering (≤5% missing nucleotides) *ITS1* host consensus sequences for additional phylogenetics analysis, we were left with *n* = 46, *n* = 26 and, *n* = 8, for genetic Clusters 1A, 1B and 2, respectively. All *ITS1* consensus sequences from our study were grouped in a single monophyletic clade with the *A. tenuis* sequence AF538489 (Figure S4). An additional *A. tenuis* reference sequence, AF538524, was more closely related to *Acropora cytherea* sequences, indicative of a possible misidentification of this sequence. Analysis of variation in *ITS1* sequences in our samples was unable to resolve Clusters 1A and 1B whereas Cluster 2 formed a monophyletic clade nested within the *A. tenuis ITS1* clade, indicating distinctness of this genetic cluster relative to Cluster 1A and 1B, which share *ITS1* variation.

**FIGURE 3:**
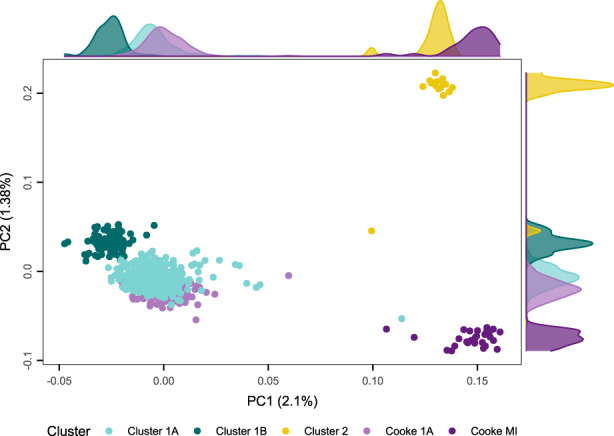
Population genetic structure of all *Acropora tenuis* samples from this study (*n* = 448) in combination with those from Cooke et al. (2020) (*n* = 148). Scatterplot illustrates the first two principal component axes summarizing genetic structure. Points are coloured with respect to genetic clusters as described in this study (refer to colour scheme in Figures 1 and 2). Cluster 1A (pale teal), Cluster 1B (dark teal) and Cluster 2 (gold) are samples from this study. Cooke 1A (pale purple) are samples from Cooke et al. (2020) that putatively belong to our defined Cluster 1A. Cooke MI (dark purple) are samples from Cooke et al. (2020) that were sampled from Magnetic Island. Density plots on the edges of the *x*‐ and *y*‐axes summarize the density distribution of samples from each cluster

Principal components analysis of 6354 SNPs (MAF > 0.5) from our data set combined with five previously published *A. tenuis* populations (Cooke et al., 2020; Figure 3) indicated shared diversity between Cluster 1A and four inshore reefs sampled between Cairns and Townsville (Fitzroy Island, Dunk Island, Pelorus Reef and Pandora Reef). A single individual from our data set sampled from Great Keppel Island in the Capricorn–Bunker Group showed an affinity to the genetically distinct Magnetic Island cluster. Although we found concordant clustering across the two data sets, regardless of filtering parameters employed, there was persistent differentiation between individuals between data sets (see Figure 3, including among individuals sampled from the same reef) indicating some subtle batch effect arising from different attributes of the data, possibly related to genomic library preparation or sequencing platforms (Lou & Therkildsen, 2022). Because of this batch effect, subsequent analyses focussed solely on data from this study.

Clustering analysis using previously published microsatellites for the 395 *A. tenuis* individuals shared between both studies did not resolve the three major genomic clusters (Figure S5). Assignment tests in the original publication found notably more geographic delineation (Lukoschek et al., 2016; Figure 5 and Figure S2) than the *STRUCTURE* results presented here. But the original study included many more colonies (*n* = 2014) and may have been able to leverage extra discriminatory power among groups as compared to a reduced data set (*n* = 365). Regardless, microsatellites both here (Figure S5) and in Lukoschek et al. (2016) failed to identify the distinct clusters revealed by thousands of SNPs (Figures 1 and 2).

### 3.3 Population genetic structure and demographic analysis among host genetic clusters

Considering only coral samples with *q* ≥ 0.85 as assigned by *NGSAdmix* as representatives of major genomic clusters (Cluster 1A, *n =* 141, Cluster 1B, *n =* 92 and Cluster 2, *n =* 14), genome‐wide average *F*_ST_ values between Cluster 2 and either Cluster 1A (0.196) or Cluster 1B (*F*_ST_ = 0.212) were substantively greater than between Cluster 1A and 1B (*F*_ST_ = 0.018). Site‐specific *F*_ST_ values varied across the genome (Figure S6); Cluster 2 was differentially fixed (*F*_ST_ ≥ 0.99) at 42 SNPs relative to Cluster 1A and at 59 SNPs relative to Cluster 1B, whereas comparisons between Clusters 1A and 1B did not indicate any fixed sites (*F*_ST_ range: 0–0.77). Within cluster differentiation was globally weak (mean weighted *F*_ST_ = 0.02) and varied between 0.01 and 0.06 among all pairwise comparisons. We found a significant association between genetic differentiation and reef distance within Cluster 1 (Mantel test *R* = 0.44, *p* = 0.01), but associations were not significant within Cluster 1B and Cluster 2 with fewer sampled populations (Figure S7).

Genome‐wide estimates of nucleotide diversity (π) were similar among Clusters 1A (mean = 0.024) and 1B (mean = 0.023), but lower for Cluster 2 (mean = 0.018; Figure S8). Similarly, estimates of average Watterson's θ per site were comparable between Cluster 1A (mean = 0.031) and Cluster 1B (mean = 0.030), which were relatively higher than Cluster 2 (mean = 0.018; Figure S8).

**FIGURE 4:**
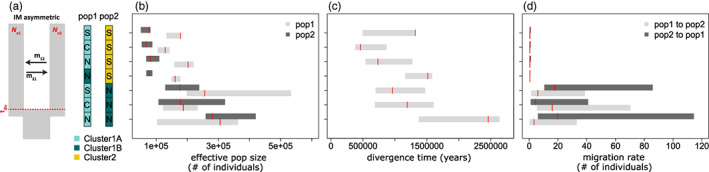
Parameter estimates for isolation with asymmetric migration for the different pairwise comparisons analysed, where isolation with asymmetric migration models were most consistently among the more likely supported scenarios. (a) Schematic of model and parameters where the coloured boxes indicate the cluster and geographic location of individuations included in the analyses with ‘N’ being from north, ‘C’ from central and ‘S’ for south. The estimates for effective population size (b), divergence time (c) and migration rates (d) were converted from *dadi* parameters following Cooke et al. (2020). Specifically, we used a mutation rate of 1.86 × 10^−8^ per base per year for the 296,667 SNPs describing the SFS to convert *θ* estimates to *N*_ref_, and a generation time of 5 years was used to convert time estimates to year. In (b) to (d), the bars indicate the 2.5%–97.5% quantile of the bootstrap estimates, while the red horizontal line shows the empirical estimate

In investigating gene flow between clusters, we explicitly compared the divergence‐only (strict isolation) model with models involving gene flow and fitted them to 2D joint‐SFS involving seven cluster pair combinations as described in the methods (see also Figure S10). The divergence model showed very poor fit for all the seven joint‐SFS comparisons that we tested, having consistently more negative log‐likelihoods (Figure S9). Among models containing gene flow, we were unable to distinguish isolation‐with‐migration (IM) scenarios (with either symmetric or asymmetric migration) against SC. The log‐likelihoods of these models exhibited a large amount of overlap. Furthermore, the estimates of demographic parameters that were shared across these models were similar (Figure S9). For example, estimates of divergence times between clusters (for IMs and SC) ranged from 0.46 to 1.5 million years (MY) with a median of ~0.8 MY after excluding the extremely high estimates obtained from comparisons of northern Cluster 1A and Cluster 1B. The estimates of SC times all occurred within 0.048–0.094 MY after the estimated divergence (except again for the estimate from northern Cluster 1A and Cluster 1B comparison); the estimated brief period of isolation in our SC model relative to the divergence potentially indicates convergence between our SC and IM results. Because of this convergence, we mainly present the results for IM with asymmetric migration (Figure 4). Thus, the most parsimonious conclusion, based on our demographic analyses, is that divergence among clusters in *A. tenuis* has likely occurred in the presence of weak and ongoing gene flow. For IM and SC models, our results suggest relatively high migration between Cluster 1A and Cluster 1B, with an estimated 1.3 to 21.6 individuals moving between clusters per generation. Indeed, actual gene flow between Cluster 1A and Cluster 1B could be greater than these estimates as our threshold cut‐off of 0.85 excluded many Cluster 1A‐like individuals (Figure S3) with secondary affinity to Cluster 1B (Figure 1). While our results indicate ongoing gene flow between Cluster 2 and both Clusters 1A and 1B, the migration rates are low, with an estimated 0.3–0.65 individuals moving between clusters per generation (Figure 4d and Figure S10). Our estimate of *N*_e_ for Cluster 2 is consistently lower than *N*_e_ estimates for Cluster 1A and Cluster 1B (Figure 4b).

### 3.4 Spatial distribution of cnidarian host genetic clusters relative to latitude and shelf position

**FIGURE 5:**
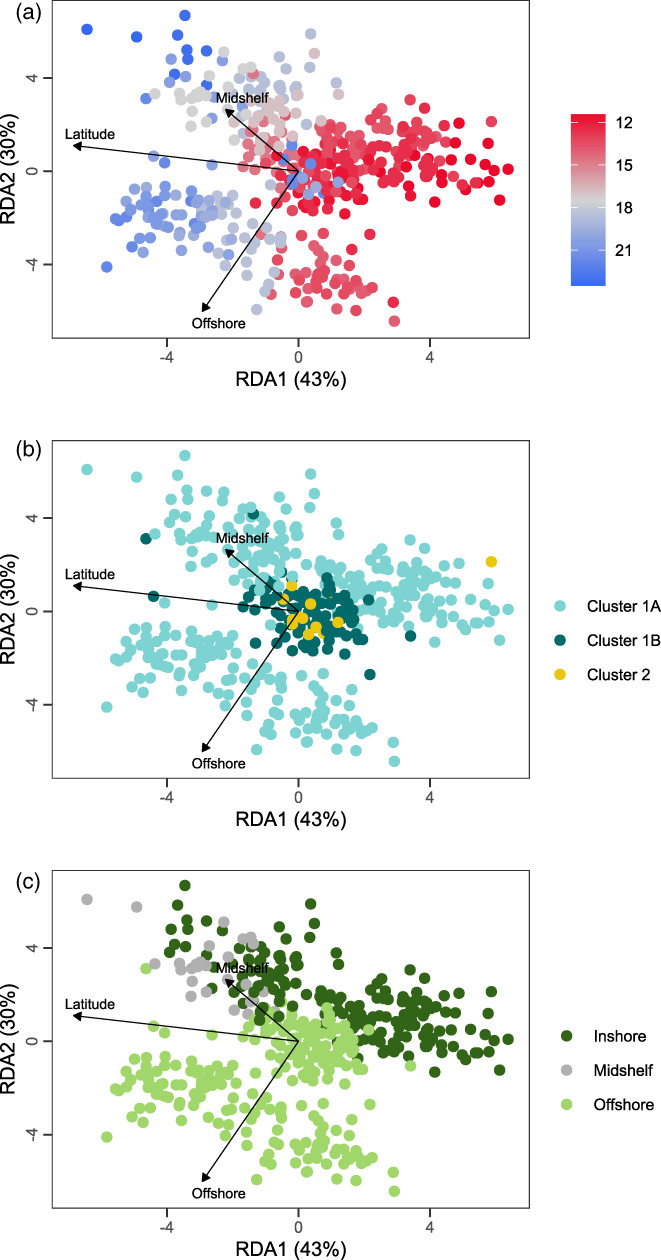
Redundancy analysis fitting coral host individual allele frequency estimates as a function of latitude and shore position. Each point represents a coral colony, coloured by (a) latitude, (b) coral host genetic cluster; and (c) shore position; see respective legends for colour keys. Arrows illustrate the relative loadings for eigenvectors corresponding to each RDA dimension. Note, both RDA1 and RDA2 significantly explained variation in the individual allele frequencies of coral hosts. Percentages in parentheses are the amount of explainable variation (0.77% of total variation) captured by each RDA dimension

The first two PC axes of the partial RDA examining spatial distribution of genotypes across the GBR described 4.7% of the total variation in individual allele frequencies (Figure 5). The full model explained significantly more variation relative to random expectations (*F*_3,442_ = 1.20, *p* = 0.001). The effect of latitude (*F*_1,442_ = 1.48, *p* = 0.001) and shore position (*F*_2,442_ = 1.05, *p* = 0.004) were both significant but small (latitude explained 0.33% of variation in individual allele frequencies, while shelf position explained 0.47%), after partialling out the effect of host genetic cluster. The effect size for spatial variables may be downwardly biased as Clusters 1B and 2 were geographically restricted; therefore, the constrained ordination was driven by samples belonging to the host genetic Cluster 1A, for which we had a large, and well distributed, number of samples. The effects of latitude and shore position on genomic variation were largely uncorrelated, as shown by the essentially perpendicular directions of effect in RDA1/RDA2 dimension space, where RDA1 accounted for 43% of the constrained variation (*F*_1,442_ = 1.52, *p* = 0.001) and RDA2 accounted for 30% of the explainable variation (*F*_1,442_ = 1.10, *p* = 0.006).

### 3.5 Assaying symbiont diversity using ITS2 and plastid genomes

Mapping coral host short reads to a set of Symbiodiniaceae *ITS2* reference sequences recovered only matches to Clade C (genus *Cladocopium*) (data from 312 colonies retained following filtering). The C40c reference matched 100% of samples, C72k in 0.64% of samples, and C72d in 0.32% of samples, with 4–275 reads mapped (mean = 17.7 per colony) to the *ITS2* locus. Having verified that symbionts were *Cladocopium*, we focussed on plastid sequence variation to describe symbiont diversity within and between coral colonies. Mapping short reads to plastids provided substantial allelic diversity and coverage (data from 223 colonies retained following filtering), with 19 loci (SNPs and indels) from two contigs (contig 1 and contig 7), with 2–17 alleles per locus. The diversity of symbiont plastid alleles within each coral colony varied from a minimum of 1, to a maximum of 8, alleles per locus, including SNPs (diallelic and triallelic) and indels. The average median read depth at a plastid locus was 86.74 reads, with a range of 26–775 median reads.

### 3.6 Spatial distribution of symbiont plastid allele diversity relative to latitude and shelf position

Spatial patterns of symbiont plastic diversity showed strong associations with geographic features. The GLM identified a significant effect of latitude (*F*_1,190_ = 12.84, *p* < 0.001), with increasing alleles per locus with increasing latitude (*β* = 0.044, *p* = 0.001). There was also a significant effect of shelf position (*F*_2,196_ = 8.32, *p* < 0.001). Relative to inshore reefs, offshore reefs had more alleles per locus (*β* = 0.097, *p* = 0.001), whereas midshelf reefs did not significantly differ from inshore reefs (*β* = 0.024, *p* = 0.728). The effect of the host genetic cluster (*F*_2,190_ = 0.81, *p* = 0.446) did not significantly predict the number of alleles per colony. Not surprisingly, there was a significant effect of read counts on the number of symbiont alleles per coral colony (*F*_1,323_ = 13.32, *p* < 0.001) and an increase in alleles per locus with increasing read counts (*β* = 0.058, *p* < 0.001). In summary, after accounting for variation in read counts, symbiont plastid allele diversity exhibited clinal variation with latitude, and lower diversity in inshore and midshelf reefs.

**FIGURE 6:**
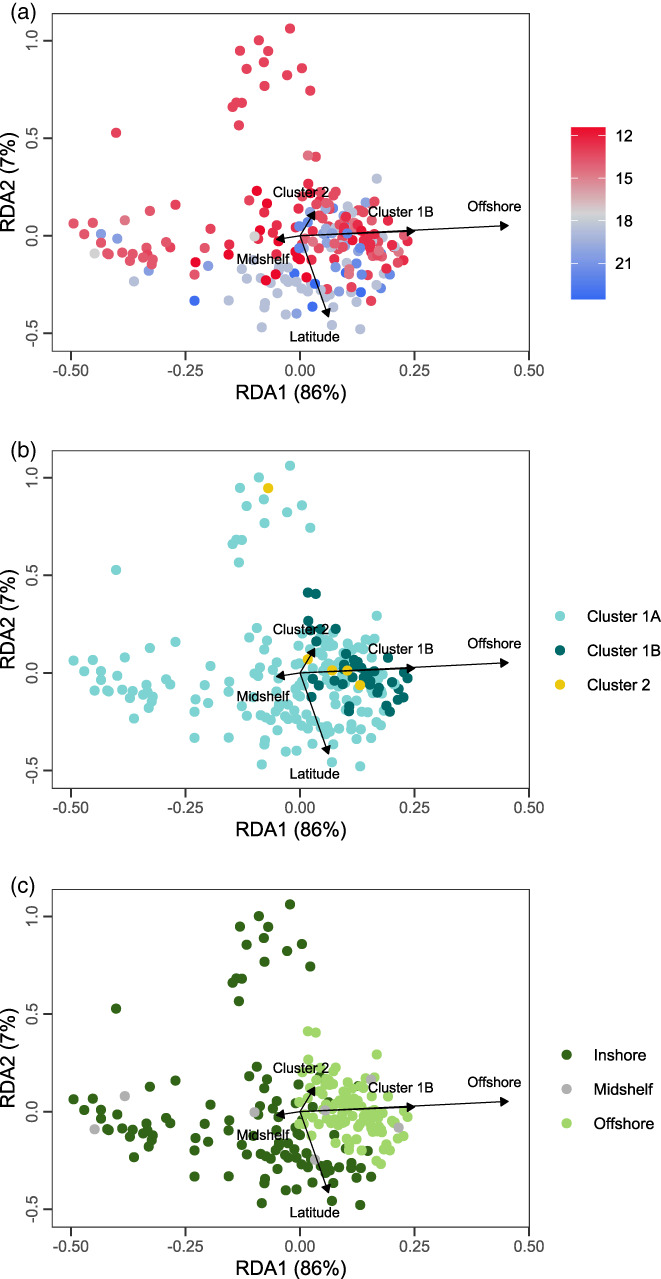
Redundancy analysis fitting symbiont allele composition as a function of latitude, coral host genetic cluster, and shore position. Each point represents a coral colony, coloured by (a) latitude, (b) coral host genetic cluster; and (c) shore position; see respective legends for colour keys. Arrows illustrate the relative loadings for eigenvectors corresponding to each RDA dimension, although only RDA1 significantly explained variation in symbiont allele composition. Percentages in parentheses are the amount of explainable variation (28.38% of total variation) captured by each RDA dimension

For the partial RDA examining how symbiont plastid allele composition was structured (Figure 6), we observed a significant effect of latitude (*F*_1,216_ = 3.83, *p* = 0.016), which explained 1.4% of variation in symbiont allele composition. The effect of shelf position was also significant (*F*_2,216_ = 24.92, *p* = 0.001) and explained 18.3% of the variation in symbiont allele composition. There was a nonsignificant effect of the host genetic cluster (*F*_2,216_ = 1.68, *p* = 0.094), which explained 1.2% of the variation in symbiont allele composition. The effect of latitude was almost completely perpendicular to the effect of the offshore factor, indicating that the effects of latitude and offshore position had largely uncorrelated effects on symbiont allele composition on the first two RDA dimensions. Only the first dimension (RDA1) was significant (*F*_1,216_ = 49.21, *p* = 0.001), accounting for 86% of the explained variation in symbiont allelic composition. Read counts accounted for 6% of variation in symbiont allele composition.

## 4 DISCUSSION

Here, we present a uniquely rich data set for a common coral species, *A. tenuis*, considering both spatial genomic diversity for the cnidarian host and associated symbiont communities across the entire GBR. We found that the cnidarian host comprised at least three well differentiated but sympatric taxa, with strong evidence for gene flow among them. Our data are consistent with growing evidence that many morphologically defined coral species comprise multiple cryptic co‐occurring taxa that are connected over evolutionary time by low‐to‐moderate levels of ongoing gene flow. Despite this differentiation in the cnidarian host, all identified *A. tenuis* taxa shared a common symbiont pool, dominated by the genus, *Cladacopium*. Rather than segregated by cnidarian host taxa, symbiont allelic variation was largely structured by shelf position, supporting the idea that broadcast spawning corals possess locally acquired and environmentally filtered endosymbiont communities. Habitat matching of coral symbionts might therefore provide an important mechanism for rapid adaptive change in corals.

### 4.1 Cryptic diversity within the *A. tenuis* host

*Acropora tenuis* is common on the GBR and throughout the Indo‐Pacific, and among the first corals to be characterized in population genomic investigations (e.g. present study; Cooke et al., 2020; Zayasu et al., 2021), complementing earlier studies based on microsatellites (Lukoschek et al., 2016; Riginos et al., 2019; Underwood, 2009). These new genomic studies all highlight cryptic divisions, largely undetected in studies with fewer loci. Among our samples, we found three distinct genetic groupings with overlapping ranges (Figures 1 and 2), and divergence times exceeding 0.5 MY (Figure 4). Hints of the three clusters were also evidenced in the coral‐based *ITS1* phylogeny, where Cluster 2 individuals formed a single monophyletic clade.

The *A. tenuis* clusters identified here complement and improve the resolution of previous differentiation based on 10 microsatellite markers (Lukoschek et al., 2016). The prior study identified genetic subdivision between southern offshore and inshore reefs and found two major groupings: (i) a widespread northern and central GBR cluster found predominantly north of 19°S and (ii) a southern offshore cluster. However, some northern reefs (e.g. No Name Reef and Lizard Island) had samples belonging to the southern cluster. This southern‐offshore versus north/central division aligns with the separation between our Clusters 1A and 1B that includes samples from No Name Reef and one individual from Lizard Island (Figure 1). Microsatellite variation in these prior studies indicated higher genetic diversity on southern reefs compared with northern reefs, likely representing the sympatric but genetically divergent clusters identified in the present study. This diversity differential provided much of the signal of north to south gene flow (Riginos et al., 2019) and will need to be re‐evaluated by restricting future gene flow estimates to same‐cluster individuals. With the present low sample numbers for Cluster 1B and Cluster 2, it is probably premature to make firm conclusions regarding their spatial distributions along the GBR.

By coanalysing genome‐wide variation from our study and from Cooke et al. (2020), we can also corroborate and extend these earlier findings for *A. tenuis*. While Cooke et al. (2020) found a distinct taxon restricted to the inshore Magnetic Island on the central GBR, our new results extend the known range for this taxon with a single sample from Great Keppel. Thus, based on the geographically extensive sample pool across both studies, there appear to be at least three genetically distinct taxa (Figures 1 and 2, and maybe four taxa: Figure 3) within what is morphologically defined as *A. tenuis*.

Although field identification of corals is challenging and can lead to cases of misidentification, our analysis of UCEs and *ITS1* sequences confirms that Clusters 1A, 1B and 2 form a monophyletic group that is synonymous with previously reported *A. tenuis* samples (Figure S4). Population genomic data and analyses are well‐suited for uncovering clusters of individuals that may have only recently stopped exchanging genes or that continue to be linked by low levels of gene flow. For instance, assignment tests (e.g. *NGSadmix*) leverage genotype compositions (assuming Hardy–Weinberg proportions and linkage equilibria within groups: Pritchard et al., 2000) to identify clusters of individuals that match expectations for unrestricted random mating. Additionally, the demographic modelling approach in *dadi* employs SFS to estimate divergence times while accounting for gene flow (Gutenkunst et al., 2009). Thus, population genomic approaches can recognize recently diverged taxa or locally adapted populations that may represent ecologically relevant diversity. In other words, the fine taxonomic resolution from population genomics (Figures 1, 2 and 4) may point to further subdivisions in *A. tenuis* than current phylogenies suggest.

### 4.2 Divergence with ongoing gene flow among genetic clusters

Hybridization and interspecific gene flow may be integral aspects of coral evolution (Hobbs et al., 2021; Willis et al., 2006). Yet, few studies have formally tested for gene flow between recent discrete taxa using demographic inference until recently (exceptions include: Cooke et al., 2020; Fifer et al., 2021; Hellberg et al., 2016; Ladner & Palumbi, 2012; Prada & Hellberg, 2020; Prata et al., 2022; Rippe et al., 2021). These historical analyses provide information on the speciation process and timing, including whether divergence occurred in the absence of gene flow (i.e. allopatric speciation) or with ongoing genetic exchange (ecological or sympatric speciation).

Our reconstructions of demographic history for *A. tenuis* show that cryptic taxa diverged prior to initial formation of the GBR and that genomic differences have been maintained despite ongoing gene flow. We found splitting times between 0.46 and 1.5 MY among all genetic clusters, despite higher genome‐wide differentiation in Cluster 2 (Figures 2 and 4). If the distinct genetic clusters do differ in distributions or habitat niches, then it is likely that modern GBR distributions reflect taxon filtering (Sommer et al., 2014) rather than adaptation in situ given these divergence times. In all cases, models of strict divergence without gene flow were rejected (Figure S9), whereas comparisons of sympatric and allopatric (nonoverlapping) populations provided replicated support for divergence with gene flow between coral taxa (Figure S10). Under the consistently highly supported isolation‐migration (IM) models, gene flow estimates were greatest between Clusters 1A and 1B (Figure 4).

Overall, our results corroborate previous estimates for late Pleistocene divergences (0.27–0.6 MY) reported between *A. tenuis* cryptic taxa (Cooke et al., 2020), albeit with older estimates in the present study. The GBR is a geologically young structure that formed in the last 0.5 MY (Chadwick‐Furman, 1996; Pandolfi & Kelley, 2008; Webster & Davies, 2003). Cyclical sea level changes during the late Pleistocene and the resulting redistribution of species ranges (Hewitt, 2000), however, likely promoted repeated SCs and periodic gene flow between coral populations. Periodic contact with pulses of historical admixture between diverging taxa may be a common evolutionary history for high dispersal marine species (e.g. Duranton et al., 2018) and could explain why we could not clearly discriminate between SC and IM models in *A. tenuis* (Figure S9). In all of our comparisons, between‐cluster migration estimates most likely reflect introgression between taxa that have some level of partial reproductive isolation. Thus, the strength or weakness of gene flow is not an accurate predictor of dispersal but likely reflects the degree of genomic permeability between taxa at different stages of the speciation process (Bierne et al., 2013; Harrison & Larson, 2016). Few fixed differences and regions of elevated differentiation in Cluster 2 comparisons (*F*_ST_ > 0.5; Figure S6) may point to parts of the genome possibly associated with reduced migration. Heterogenous divergence across genomes, however, between taxa may result from a wide range of underlying molecular processes (Cruickshank & Hahn, 2014; Ravinet et al., 2017). Determining whether regions of elevated differentiation are related to differential introgression, the origins of those alleles (e.g. Rose et al., 2021), and whether they help maintain local adaptation in high dispersal *Acropora* corals will be outstanding questions for understanding the processes shaping coral diversity.

### 4.3 Geographic gradients and associated genetic diversity of hosts and symbionts

In *A. tenuis*, we found that both cnidarian host genotypes and symbiont allelic variation were structured with respect to latitude and shelf position. For the cnidarian host, latitude and shelf position together explained a very small but significant proportion of variation (0.77%) in individual allele frequencies. This signal was largely driven by Cluster 1A for which we had the most widely distributed samples (Figure 5). Broader sampling of Clusters 1B and 2 might reveal similar patterns, but it is also possible that these clusters are subject to greater environmental forcing than Cluster 1A, leading to restricted distributions (Sommer et al., 2014).

In contrast, genetic differentiation by latitude and shelf position was substantial for symbionts, explaining 19.7% of variation in compositional turnover (Figure 6). *ITS2* sequences showed that all three cnidarian host clusters were dominated by symbionts from the genus *Cladocopium* (100%). All coral colonies contained the C40c strain, and <1% contained C72k and C72d. Yet, by recovering plastid sequences from colony‐level shotgun sequencing, we could further characterize allelic variation and test the effects of the environment on symbiont structure. Our novel method of describing symbiont diversity based on plastid derived alleles appears to yield more information than strain identity based on *ITS2* sequences. Because genomic sequencing is typically obtained from samples of the coral holobiont, our approach demonstrates the great potential of using cocaptured symbiont genome sequences to study variation variations in these endosymbiotic bacteria alongside their cnidarian hosts.

Allelic diversity of *Cladocopium* plastids varied by shelf position, and to a lesser extent by latitude, both in terms of alpha (number of alleles within colonies) and beta (change in allelic composition between colonies) diversity. These distinct aspects of spatial diversity were evidenced in the GLM results that focussed on spatial patterns of within‐colony diversity. Colonies from northern and further offshore sites had more plastid alleles per locus, compared with colonies from southern, inshore and midshelf locations. Our RDA of symbiont allelic composition showed substantial turnover along latitudinal and shelf gradients (Figure 6). Symbiont plastid diversity or differentiation showed no relationship to host genetic cluster in either GLM or RDA analyses. While coral‐symbiont associations may show strong evolutionary specificity (Prada et al., 2014), our observations support the notion that the specificity and maintenance of coral‐*Symbiodinium* symbiosis can also be influenced by environmental gradients (Tonk et al., 2013) more so than related host taxa.

Our observations that *A. tenuis* symbionts show strong environmental filtering and are not associated with host structure corroborate findings from Cooke et al. (2020). In their study, *Cladocopium* also was the dominant genus, and *Cladocopium* mitochondrial haplotypes were associated with exposure to riverine plumes, whereas cnidarian host variation was not correlated with freshwater inputs. Those colonies most heavily exposed to riverine plumes shared a single *Cladocopium* mitochondrial haplotype, consistent with our finding of lower symbiont diversity for inshore *A. tenuis* colonies. Lower diversity has also been observed among inshore soft corals on the GBR (Howells et al., 2009). It is possible that the inshore–offshore habitat gradient represents a ubiquitous environmental filter for symbionts across coral species. Additionally, our data from *A. tenuis* suggest that whilst conditions faced by inshore colonies decreases local symbiont diversity (i.e. reduced alpha diversity), greater environmental variance might promote variation among inshore locations (i.e. increased beta diversity).

Our study focussed on symbiont diversity in *A. tenuis* at the scale of the GBR. However, symbiont strains can exhibit significant turnover across very short physical distances, for example, between leeward and windward sides of coral islands (Howells et al., 2013), and symbiont composition often exhibits significant turnover with respect to depth (Bongaerts et al., 2010; Prada et al., 2014; van Oppen et al., 2018; Warner et al., 2015). Local variation in symbiont composition and diversity has been also linked to environmental adaptation (such as, Rose et al., 2021). Therefore, joint considerations of both coral host and symbiont spatial genetic patterns can help identify the relevant environmental factors and spatial scales of differentiation that are likely to reflect adaptation to local conditions. Understanding the congruence or mismatch in spatial genetic patterns between coral host and symbionts is particularly important today as they provide insights into how adaptive variation is spatially configured, thereby providing information for spatial planning (Riginos & Beger, 2022), restoration (Baums et al., 2019) and assisted management (van Oppen et al., 2017) in light of climate change and other anthropogenic stressors.

### 4.4 Challenges in combining genomic data sets

In principle, combining genomic data sets from different studies offers incredible potential to increase spatial replication or investigate temporal shifts in population genetic patterns. Given that taxonomic relationships among corals are sometimes contentious and field identification to the species level can be challenging, it is especially important (in our opinion) that investigators try to build explicit links between new and previously published DNA sequence‐based data sets. Throughout this investigation, we have endeavoured to place our new results in the context of previous work on *A. tenuis*, including downsampling our data to allow phylogenetic analyses (Figure S4), and connecting to previous microsatellite results (Figure S5).

Genomic data sets from different studies, however, can thwart amalgamation. Here, we were particularly interested in merging our *A. tenuis* samples with those from a low‐coverage whole‐genome study by Cooke et al. (2020). Coanalysing these data required a balance between aggressive filtering versus retaining sufficient loci to extract subtle patterns. Despite our best efforts, weak study (batch) effects emerged, differentiating samples from this study and those from Cooke et al. (2020) (see slight separation between individuals from both studies: Figure 4). Batch effects are common in population genomic investigations (Tom et al., 2017) and might be particularly problematic in comparisons between studies using low‐coverage genomic sequencing (Lou & Therkildsen, 2022). This challenge in unifying results across studies is worrisome as the value of individual studies is diminished if they cannot be contextualized against earlier results. Fine‐tuning quality metrics and masking genomic regions subject to batch effects or resequencing previously published samples alongside new data sets might provide solutions for identifying and resolving batch effects (Tom et al., 2017). As more studies move to whole‐genome sequencing, methods may also evolve to resolve this issue. We are confident that the overall filtering of our *A. tenuis* samples is sufficient to identify the major genetic clusters in this study and those shared with Cooke et al. (2020). Nonetheless, we did not pursue additional analyses on a merged data set because of suspected batch effects.

### 4.5 Cryptic taxonomic diversity and implications for coral conservation

Delineating the natural groupings of frequently interbreeding individuals provides critical information for conservation and management (Hey et al., 2003). Although species delimitations are seemingly easier to understand, they may belie the true scope of ecologically and evolutionarily relevant diversity. Our findings add to a growing number of studies that highlight how current taxonomic hypotheses (Cowman et al., 2020) underestimate diversity in reef‐building corals, especially within the diverse Acroporid clade. These results indicate that inferences based on species distributions—such as range size, thermal tolerance, habitat usage and other niche attributes—are questionable without first documenting the range extents and niches of the distinct cryptic taxa. For instance, commonly used conservation planning schemes based on optimizing species representation necessitate knowing which species live where (reviewed by, Riginos & Beger, 2022). Without better documentation on the distributions of cryptic coral species, we lack the information to accurately map species ranges and therefore to formulate conservation plans seeking to preserve coral biodiversity.

Conservation planning for future reefs is likely to prioritize coral species that can withstand high temperatures (Mumby et al., 2010). But cryptic species can differ in thermal physiology. Extensive studies of cryptic and sympatric *A. hyacinthus*, for example, highlight extensive differences in physiological responses (reviewed by Thomas et al., 2018) and identify specific genomic regions that may contribute to the cryptic taxa's distinctive bleaching susceptibility (Rose et al., 2021). Similar results are suggested for *Pocillopora* (Burgess et al., 2021) and *Porites* (Boulay et al., 2014), where cryptic taxa differ in survival through bleaching events. Again, uncertainty regarding cohesive genetic species can lead to erroneous biological inferences (Gomez‐Corrales & Prada, 2020) and therefore potentially inappropriate conservation recommendations.

Genomic tools are starting to provide windows into the complex ecological and evolutionary dynamics of coral holobionts in relation to geography and environment. The common finding that low levels of gene flow connect divergent taxa, however, may provide opportunities for adaptation and could contribute to the future well‐being of coral reefs. Several examples in diverse taxa point to intraspecific gene flow as an important aspect of local adaptation (Huerta‐Sánchez et al., 2015; Oziolor et al., 2019), and the geographically widespread nature of gene exchange (Figure 4) in *A. tenuis* suggests that universally adaptive alleles should cross taxon boundaries. In *A. tenuis*, zooxanthellate endosymbionts appear to easily traverse host taxa, providing yet another distinct aspect of adaptive interchange, likely allowing coral populations to respond to environmental stressors on ecological timescales. These emerging findings may guide future management strategies that aim to preserve ecological and evolutionary functions including adaptation capacity.

## CONFLICT OF INTEREST

The authors have no conflict of interest to declare.

## DATA ACCESSIBILITY STATEMENT

Genetic data: Raw sequence reads are deposited in the SRA (BioProject PRJNA849642) and individual genotype data are available on Data Dryad (48096232EVA‐2022‐007‐OA.R1). Sample metadata can be found in GEOME (http://n2t.net/ark:/21547/EFV2; including georeferences in decimal degrees and year of sampling event).

## BENEFIT‐SHARING STATEMENT

This research collaboration involves collaborators from Australian institutions where all collaborators are included as co‐authors. The results of research have been shared with the provider communities and the broader scientific community (see above), and the research addresses a priority concern, in this case the conservation of corals. Benefits from this research accrue from the sharing of our data and results on public databases as described above.

## Supporting information


Appendix S1

## Data Availability

Raw data can be found on NCBI's Short Read Archive, submission SUB11600324, where geographical metadata are deposited on GEOME (http://n2t.net/ark:/21547/EFV2). VCF files and supporting scripts are on Data Dryad (48096232EVA‐2022‐007‐OA.R1).
