# Supplementary material for: Cryptic diversity and spatial genetic variation in the coral Acropora tenuis and its endosymbionts across the Great Barrier Reef
Source: Evol Appl. 2022 Jul 7;16(2):293–310. doi: 10.1111/eva.13435 (PMC9923489; doi:10.1111/eva.13435)
Supplement: Supplementary file 1 — Appendix S1 [file EVA-16-293-s001.zip › eva13435-sup-0001-FiguresS1-S10.pdf]

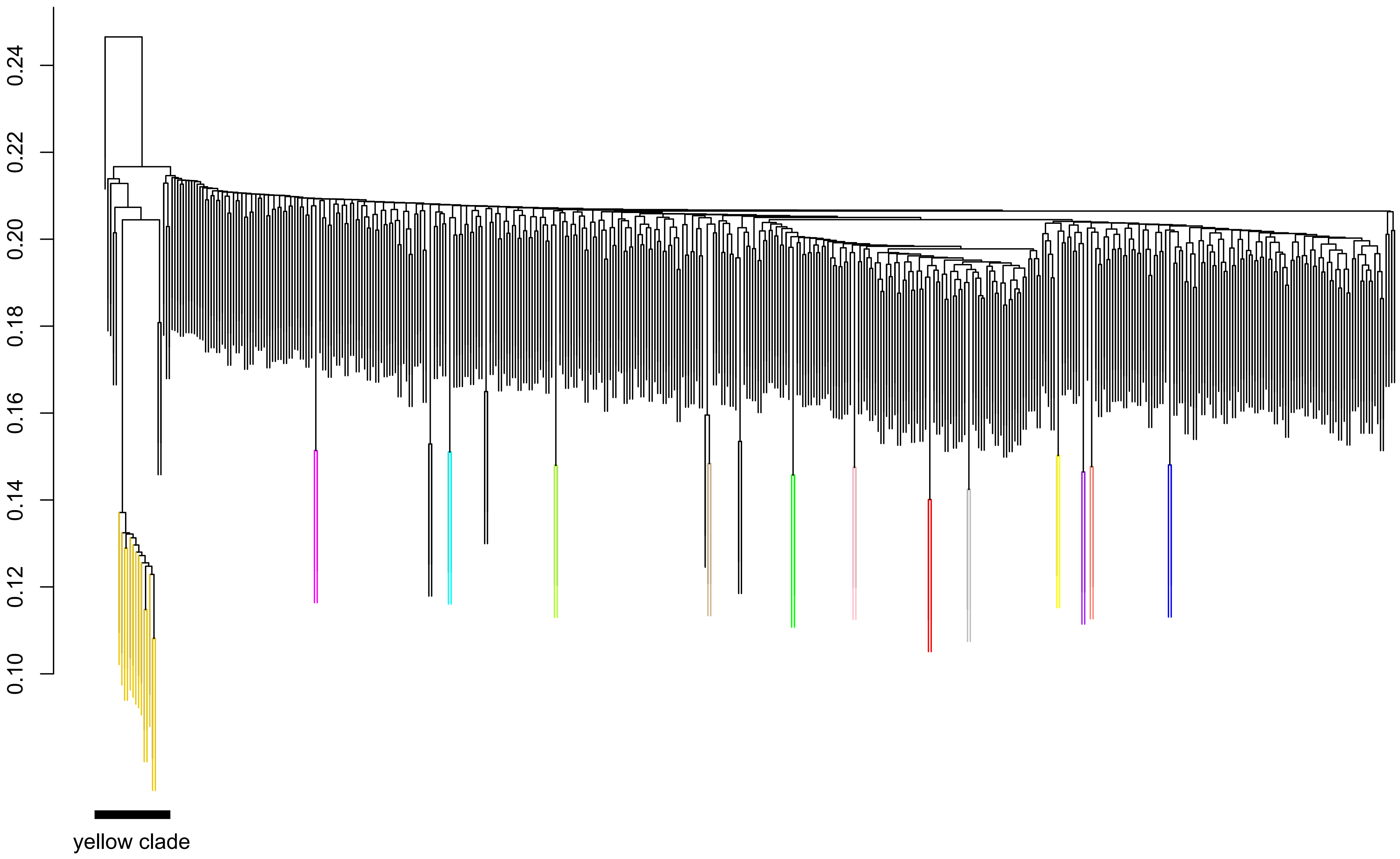
0.24
0.22
0.20
0.18
0.16
0.14
0.12
0.10
yellow clade

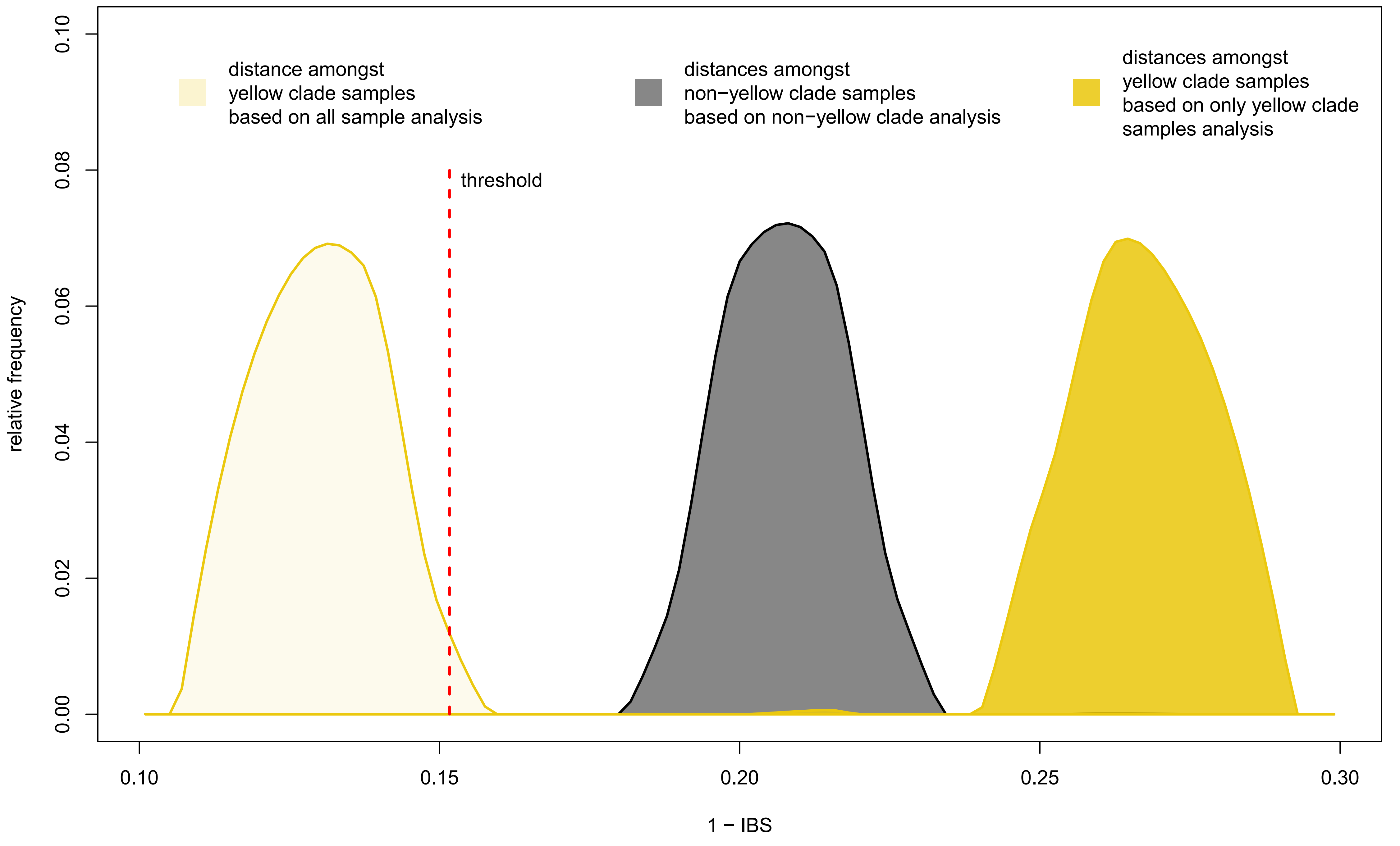

distance amongst
yellow clade samples
based on all sample analysis
distances amongst
non−yellow clade samples
based on non−yellow clade analysis
distances amongst
yellow clade samples
based on only yellow clade
samples analysis
threshold
relative frequency
0.00
0.02
0.04
0.06
0.08
0.10
0.10
0.15
0.20
0.25
0.30
1 − IBS

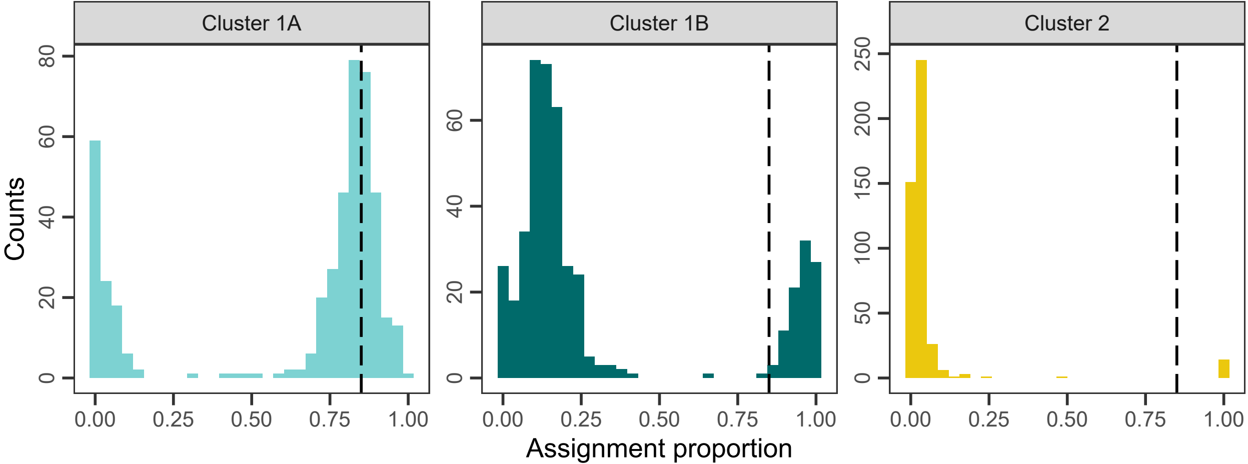

Cluster 1A
Cluster 1B
Cluster 2
Counts
Assignment proportion
0
20
40
60
80
0
20
40
60
0
50
100
150
200
250
0.00
0.25
0.50
0.75
1.00

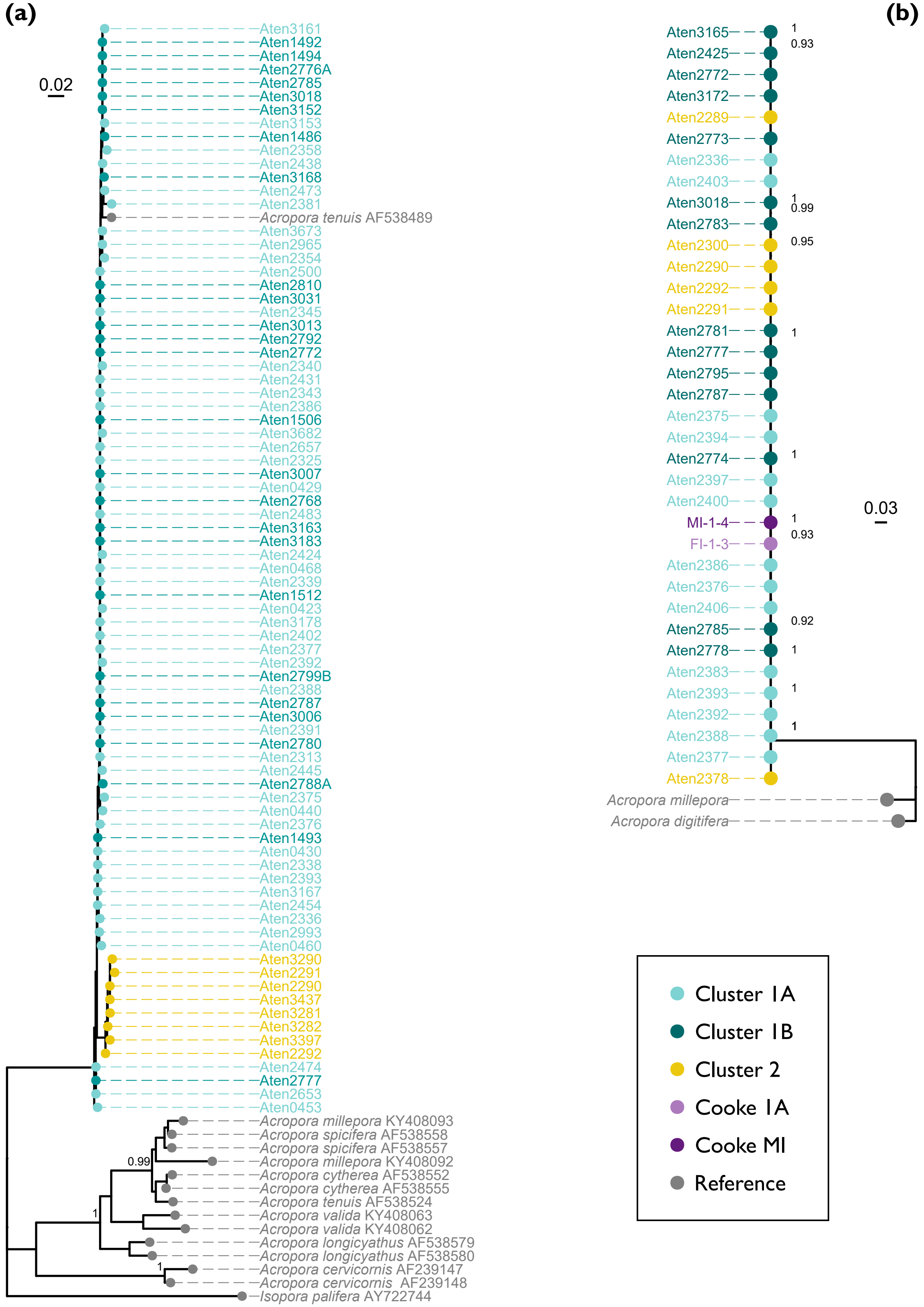
(a)
0.02
Aten3161
Aten1492
Aten1494
Aten2776A
Aten2785
Aten3018
Aten3152
Aten3153
Aten1486
Aten2358
Aten2438
Aten3168
Aten2473
Aten2381
Acropora tenuis AF538489
Aten3673
Aten2965
Aten2354
Aten2500
Aten2810
Aten3031
Aten2345
Aten3013
Aten2792
Aten2772
Aten2340
Aten2431
Aten2343
Aten2386
Aten1506
Aten3682
Aten2657
Aten2325
Aten3007
Aten0429
Aten2768
Aten2483
Aten3163
Aten3183
Aten2424
Aten0468
Aten2339
Aten1512
Aten0423
Aten3178
Aten2402
Aten2377
Aten2392
Aten2799B
Aten2388
Aten2787
Aten3006
Aten2391
Aten2780
Aten2313
Aten2445
Aten2788A
Aten2375
Aten0440
Aten2376
Aten1493
Aten0430
Aten2338
Aten2393
Aten3167
Aten2454
Aten2336
Aten2993
Aten0460
Aten3290
Aten2291
Aten2290
Aten3437
Aten3281
Aten3282
Aten3397
Aten2292
Aten2474
Aten2777
Aten2653
Aten0453
Acropora millepora KY408093
Acropora spicifera AF538558
Acropora spicifera AF538557
0.99
Acropora millepora KY408092
Acropora cytherea AF538552
Acropora cytherea AF538555
Acropora tenuis AF538524
1
Acropora valida KY408063
Acropora valida KY408062
Acropora longicyathus AF538579
Acropora longicyathus AF538580
1
Acropora cervicornis AF239147
Acropora cervicornis AF239148
Isopora palifera AY722744
(b)
Aten3165
1
0.93
Aten2425
Aten2772
Aten3172
Aten2289
Aten2773
Aten2336
Aten2403
Aten3018
1
0.99
Aten2783
Aten2300
0.95
Aten2290
Aten2292
Aten2291
Aten2781
1
Aten2777
Aten2795
Aten2787
Aten2375
Aten2394
Aten2774
1
Aten2397
Aten2400
MI-1-4
1
0.93
FI-1-3
Aten2386
Aten2376
Aten2406
Aten2785
0.92
Aten2778
1
Aten2383
Aten2393
1
Aten2392
Aten2388
1
Aten2377
Aten2378
Acropora millepora
Acropora digitifera
0.03
Cluster 1A
Cluster 1B
Cluster 2
Cooke 1A
Cooke MI
Reference

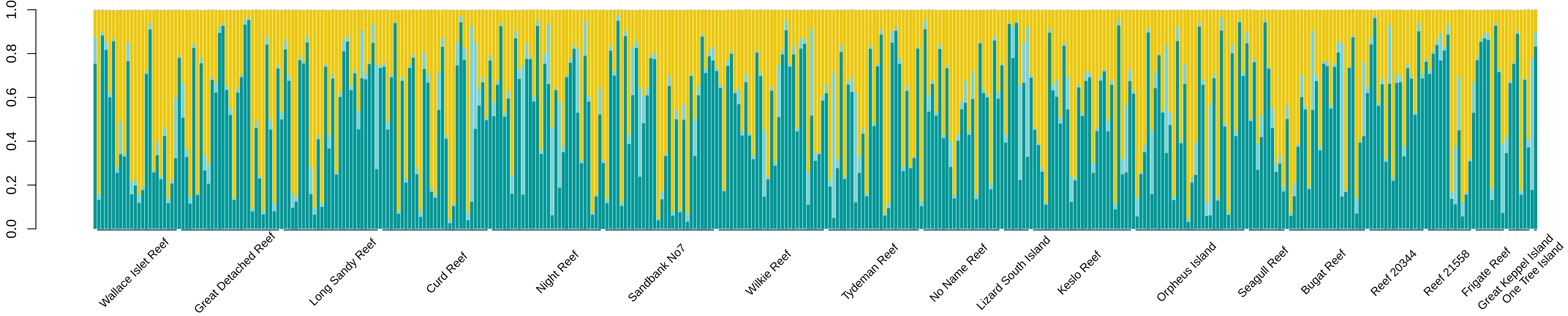

0.0
0.2
0.4
0.6
0.8
1.0
Wallace Islet Reef
Great Detached Reef
Long Sandy Reef
Curd Reef
Night Reef
Sandbank No7
Wilkie Reef
Tydeman Reef
No Name Reef
Lizard South Island
Keslo Reef
Orpheus Island
Seagull Reef
Bugat Reef
Reef 20344
Reef 21558
Frigate Reef
Great Keppel Island
One Tree Island

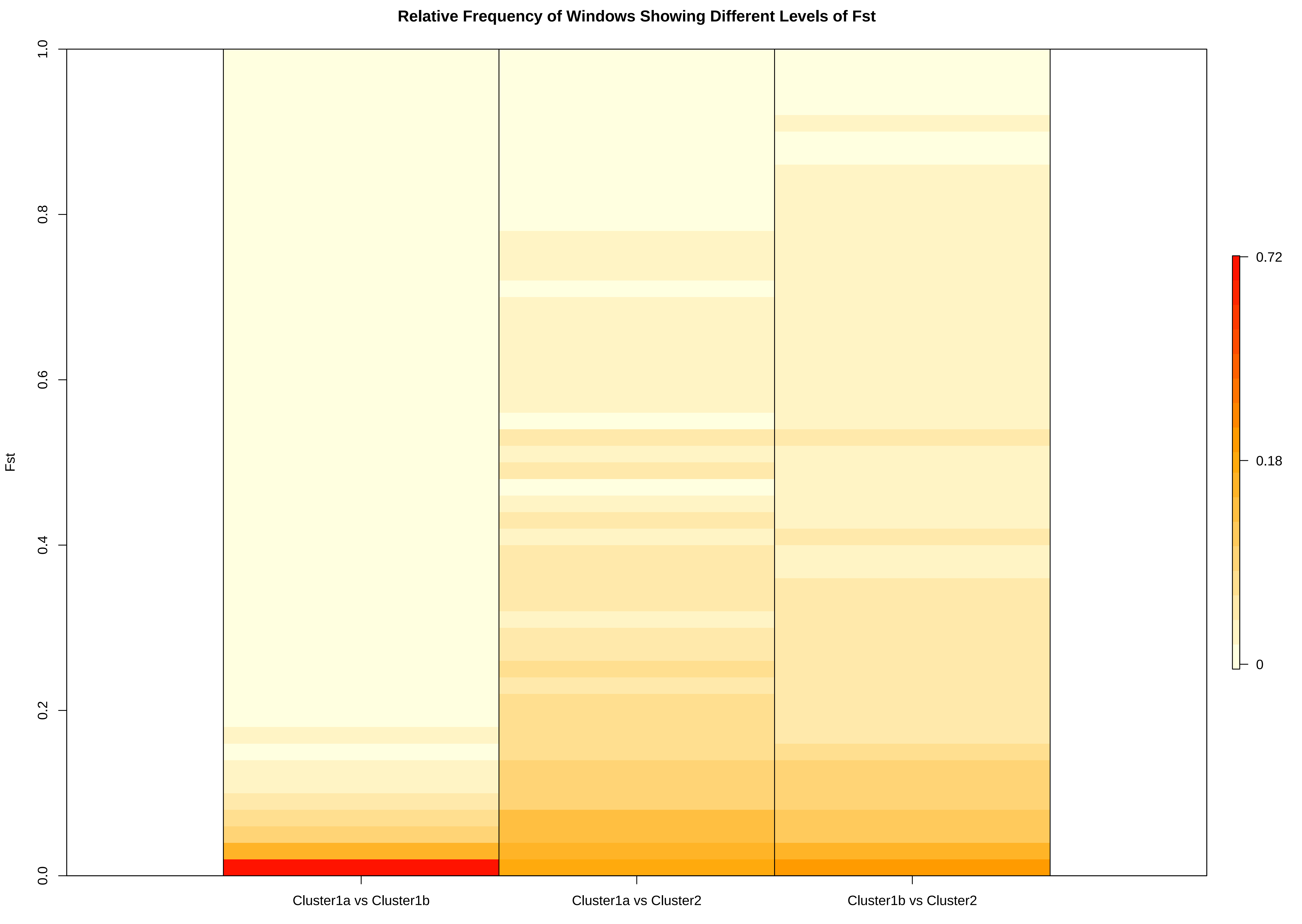

Relative Frequency of Windows Showing Different Levels of Fst
Fst
1.0
0.8
0.6
0.4
0.2
0.0
Cluster1a vs Cluster1b
Cluster1a vs Cluster2
Cluster1b vs Cluster2
0.72
0.18
0

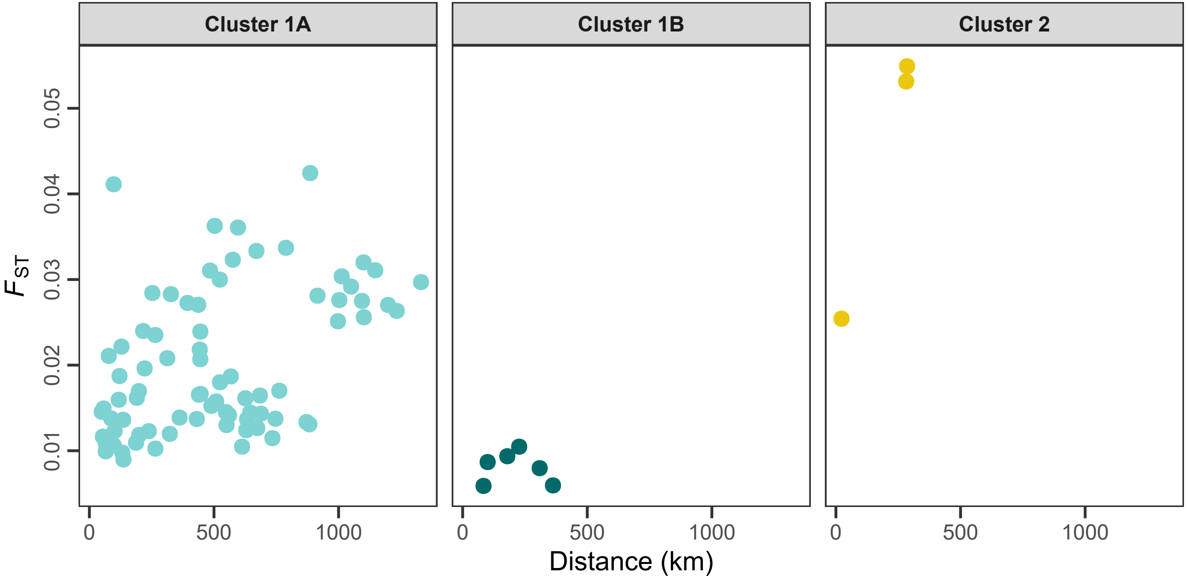
Cluster 1A
Cluster 1B
Cluster 2
F_ST
0.01
0.02
0.03
0.04
0.05
0
500
1000
Distance (km)

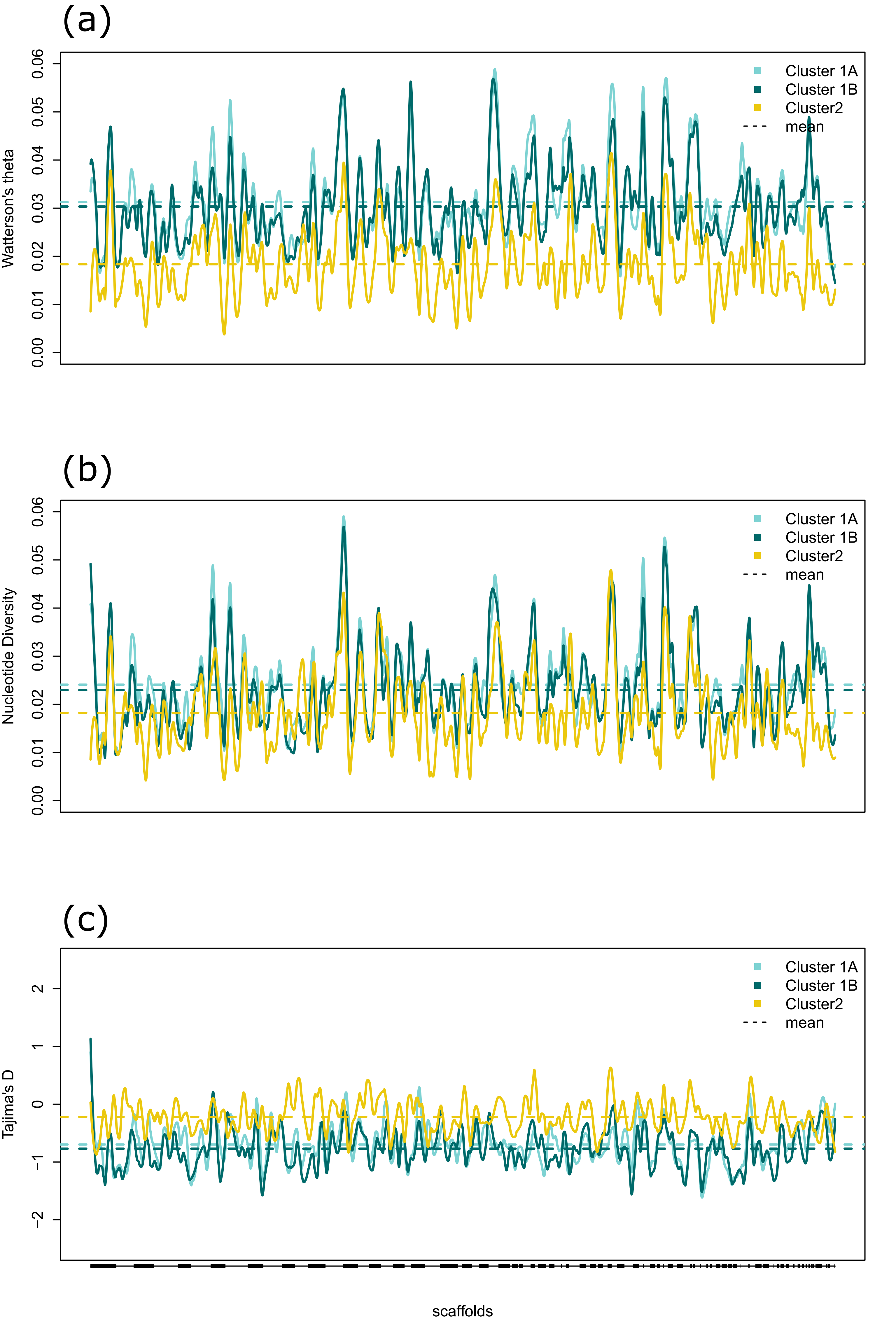
(a)
Watterson's theta
0.00
0.01
0.02
0.03
0.04
0.05
0.06
Cluster 1A
Cluster 1B
Cluster2
mean
(b)
Nucleotide Diversity
0.00
0.01
0.02
0.03
0.04
0.05
0.06
Cluster 1A
Cluster 1B
Cluster2
mean
(c)
Tajima's D
−2
−1
0
1
2
Cluster 1A
Cluster 1B
Cluster2
mean
scaffolds

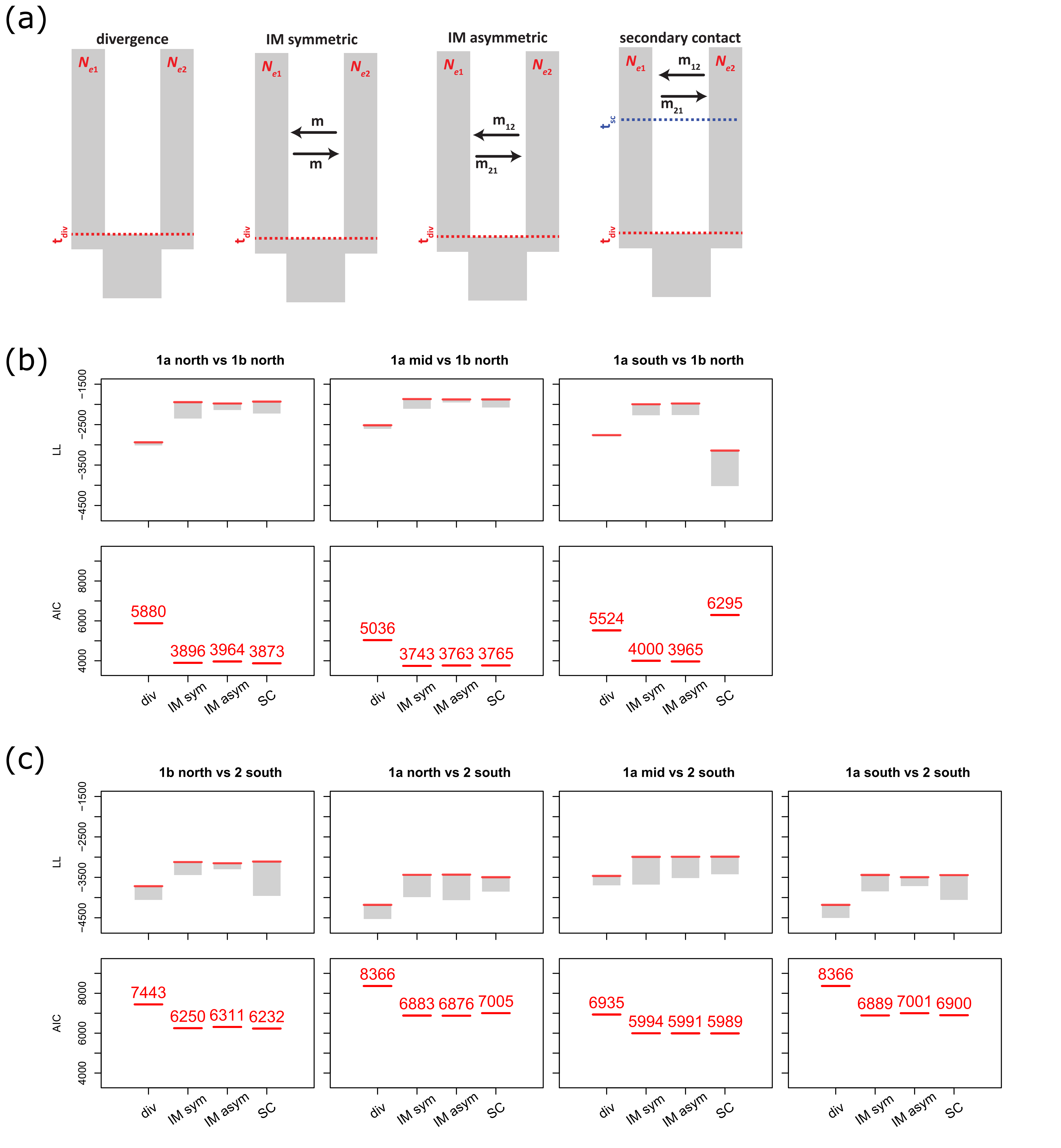
(a)
divergence
IM symmetric
IM asymmetric
secondary contact
Ne1
Ne2
m
m12
m21
tdiv
tsc
(b)
1a north vs 1b north
1a mid vs 1b north
1a south vs 1b north
LL
AIC
5880
3896 3964 3873
5036
3743 3763 3765
5524
4000 3965
6295
div
IM sym
IM asym
SC
(c)
1b north vs 2 south
1a north vs 2 south
1a mid vs 2 south
1a south vs 2 south
7443
6250 6311 6232
8366
6883 6876 7005
6935
5994 5991 5989
8366
6889 7001 6900

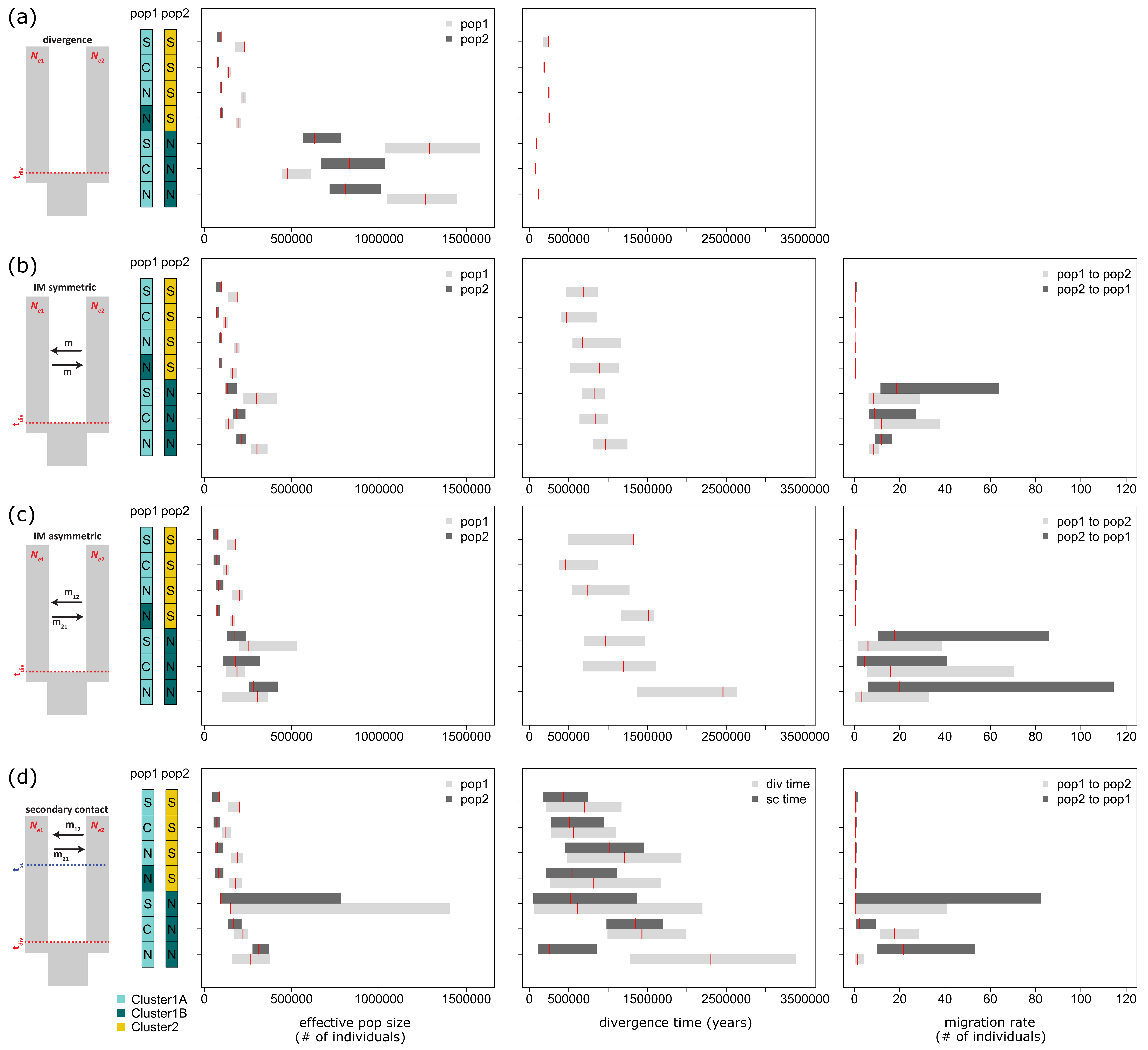
(a)
divergence
pop1 pop2
(b)
IM symmetric
(c)
IM asymmetric
(d)
secondary contact
pop1
pop2
div time
sc time
pop1 to pop2
pop2 to pop1
Cluster1A
Cluster1B
Cluster2
effective pop size
(# of individuals)
divergence time (years)
migration rate
(# of individuals)
