# Supplementary material for: Cryptic diversity and spatial genetic variation in the coral Acropora tenuis and its endosymbionts across the Great Barrier Reef
Source: Evol Appl. 2022 Jul 7;16(2):293–310. doi: 10.1111/eva.13435 (PMC9923489; doi:10.1111/eva.13435)
Supplement: Supplementary file 1 — Appendix S1 [file EVA-16-293-s001.zip › eva13435-sup-0002-Supinfo.pdf]

SUPPLEMENTARY MATERIALS

SUPPLEMENTARY MATERIALS AND METHODS

DNA extraction and genomic library preparation

Genomic DNA was extracted from 25mg of coral tissue following a modified version of a salt extraction method (Bongaerts et al., 2017; Wilson et al., 2002). Whole genome libraries were prepared at the Australian Genome Research Facility (AGRF, Melbourne Australia) using the Illumina Nextera library preparation kit with no size selection.

We developed custom sequence probes to enhance high coverage of randomly selected loci across the *A. tenuis* genome. A random sample of nuclear SNPs were chosen from the *A. tenuis* draft genome and population genomic dataset (aten_final_0.11.fasta; Cooke et al. 2021) after passing quality criteria (Table S1). The final dataset of targeted regions consisted of 22,908 loci containing the focal SNP and 50 bp on either side of the variant (100 bp total length). Custom 80 bp probes were designed and synthesized by Arbor Biosciences (formerly MYcroarray; Ann Arbor, MI). The final bait set consisted of 40,000 probes targeting 21,304 loci that passed Arbor quality criteria, with two probes overlapping every 40 nucleotides to achieve 2x tiling over targeted loci. Target enrichment was performed at AGRF following the manufacturer's MyBaits-2 protocol. A total of 96 hybridisation reactions were performed on sets of 16 multiplexed samples at equimolar concentrations. We performed shallow (5x) sequencing on 698 colonies using four lanes of an Illumina HiSeq HT (125bp paired end reads). In all, 698 individual colonies were sequenced along with 4 technical replicates (Table S2).

Genomic data processing, filtering, and clone identification

We used FastQC (http://www.bioinformatics.bbsrc.ac.uk/projects/fastqc) to examine read quality and adapter contamination. Raw reads were filtered for a minimum quality phred-score of ≤ 10 and a minimum length of 80 bp. Adapter sequences and reads that are likely sequencing artefacts (e.g., phiX) were removed using bbduk of the bbtools package (Bushnell, 2014). Paired reads were mapped against the *Acropora tenuis* draft genome (aten_final_0.11.fasta; (Cooke et al., 2020) using BWA and the MEM algorithm with default parameters (Li & Durbin, 2009). The resulting alignments (SAM files) were converted to

binary BAM files, which were indexed and sorted using Samtools vX (Li et al., 2009). We added read groups to individual BAM files and marked PCR duplicates using picard (http://broadinstitute.github.io/picard/). For samples that were re-sequenced, the BAM files were merged – and thus, giving one BAM file for each sample.

We first used ANGSD (Korneliussen, Albrechtsen, & Nielsen, 2014) to restrict our dataset to the highest coverage individuals and genome-wide loci for downstream analyses. We estimated absolute reads counts (-doCounts) for each individual across all sites, including baited and non-baited regions passing minimum quality criteria of mapping quality 30 and base quality 30 (-minMapQ 30 -minQ 30). We retained only sites covered by at least 3 reads in >= 40% of individuals based on absolute reads counts. We then selected only the top 65% of individuals ranked by the total number of positions covered by at least 3 reads. The final high-quality dataset resulted in 463 individuals and 296,667 loci falling within baited and non-baited genomic regions that met our coverage criteria (Table S2). Unfortunately, nearly all individuals from the Capricorn Bunker group (represented by Heron Island and One-Tree Island) were removed due to low quality DNA.

We identified potential clones among our samples following the approach of Manzello et al. (2019). We used ANGSD to estimate identity-by-state (IBS) between individuals, using the single read re-sampling approach, which is robust to read depth variation across individuals, where IBS is the proportion of randomly sampled reads covering a polymorphic site that are identical between compared individuals. The sites used for this analysis required minimum filtering criteria of mapping quality 30, base quality 30, coverage >= 3 reads in 85% of individuals. We called major and minor alleles directly from the genotype likelihoods assuming biallelic sites, where the major allele was the most frequent (-doMajorMinor 1) and considered only polymorphic with a likelihood ratio test p-value <0.000001. We termed these specific quality criteria as "Q15" (Quality with ≤15% missing data). For IBS analyses, we additionally applied a minimum minor allele frequency of 0.05. The resulting output provided estimated pairwise genetic differences between samples (i.e., 1 - IBS). We made use of four pairs of technical sequence replicates to determine the minimum similarity threshold for selecting multilocus genotypes (MLG). Specifically, we used the upper 95% confidence of the mean distances between sequence replicates. Clones were identified as sets of individuals whose differences fell below this threshold. We first estimated IBS including all samples that had passed filtering (n=463, including technical

replicates and MLGs). Applying the approach of Manzello et al. (2019) as described above, however, resulted in all samples belonging to the most genetically distant clade being identified as clones (Fig. S1, i.e., yellow clade). Because it is unlikely that all samples from these differentiated clades are clones, we generated separate IBS matrices for only samples from the yellow clade (n=14), and another one including all other samples (n=449). We then performed the analysis individually on each group to identify potential clones. As expected, performing the IBS per major clade resulted in high genetic distance (1-IBS) between differentiated samples (i.e., yellow clade) previously identified as potential clones (Fig. S2).

Identification of major host clusters, verification of taxonomy, and integration with previous Acropora tenuis studies

Cluster identification was based on *PCAngsd* (Meisner & Albrechtsen, 2018) and *NGSadmix* (Skotte, Korneliussen, & Albrechtsen, 2013). Genotype likelihoods were estimated in *ANGSD* using the samtools model (-gl 1). We applied our Q15 filtering criteria as described above and output genotype likelihood estimates in Beagle-format (-doGlf 2; (Browning & Browning, 2007). To evaluate population genetic structure, we extracted an individual covariance matrix using *PCAngsd* and applied a 0.05 minor allele frequency (MAF) threshold. We then computed eigenvectors and eigenvalues in R (R Development Core Team 2019) using the function eigen() and performed a Principal Components Analysis (PCA). We used a Bayesian hierarchical clustering method in *NGSadmix* to estimate individuals ancestry proportions assuming 2-6 ancestral populations (K genetic clusters) applying a MAF threshold of 0.05. To contextualise the taxonomic relationships of these clusters with other *Acropora* species, we identified a subset of samples that were representative of their genetic clusters (assignment probability ≥ 0.85 obtained through *NGSadmix*). The 0.85 threshold represents a natural breakpoint that delineated individuals comprising Clusters 1B and 2, but many Cluster 1a-like individuals fell below the 0.85 threshold, perhaps indicative of greater admixture in this group (Fig. S3). However, the analyses that follow strive to understand relationships among putative parental taxa, so focusing on the most likely Cluster 1a parental types is appropriate. A bias that this threshold may bring is an underestimation of intercluster gene flow involving Cluster 1a.

To combine datasets based on UCE and exon sequences we undertook an *in silico* target-capture and locus assembly processes. First we mapped the hexa-v2 probe sequences (Cowman et al., 2020) to the *A. tenuis* (http://aten.reefgenomics.org/), *A. millepora* and *A.*

digitifera (https://www.ncbi.nlm.nih.gov/assembly/GCA_014634065.1/) genomes using bwa mem (0.7.17; (Li, 2013), retaining only primary alignments and then removing any alignments with more than 20 hard or soft clipped bases with samclip (0.4.0; https://github.com/tseemann/samclip). Using bedtools (2.30.0; (Quinlan & Hall, 2010)) we then identified a 1000bp region around each probe (500bp up and downstream of the central aligned base) and merged overlapping regions to create a set of intervals for each species representing assembled loci. We then extracted a subset of aligned reads overlapping these intervals from bam files previously generated for a representative set of 38 of our *A. tenuis* samples with the greatest number of reads along with two samples from Cooke et al. (2020) from Fitzroy Island and Magnetic Island respectively. We then called consensus sequences for each individual using bcftools (1.11; (Li, 2011) representing heterozygous sites using the appropriate IUPAC ambiguity code. Bedtools was then used to extract sequences for each UCE/Exon locus per individual from these consensus sequences and from each of the reference genomes, included as additional individuals. This initial set of UCE sequences was then filtered to include only those found in all three species, resulting in a final set of 1699 common loci. Alignments for each locus were generated using mafft (7.394; (Katoh & Standley, 2013) with settings for high accuracy '--maxiterate 1000 --localpair' and to allow for sequences on opposite strands. Resultant alignments were used for phylogenetic inference with IQ-Tree (2.1.2; (Minh et al., 2020) in two steps. In the first step, each locus was placed into its own partition and partition finder was used with the `MF+MERGE` setting to generate an optimised set of partitions and model for each partition. In the second step, this optimal partition scheme was used with 1000 ultrafast bootstraps to infer the final phylogeny (Fig. S4).

To enable phylogenetic analyses based on ITS1, the location of the *ITS1* gene was identified by mapping a GenBank-sourced *Acropora tenuis ITS1* sequence (AF538489) to our reference genome with bwa mem. *ITS1* Alignments in our samples were combined using *bcftools mpileup* and variants were called using *bcftools* call. Indels were normalized with *bcftools norm* and BAM files were indexed with *bcftools index*. A consensus sequence for each specimen was called with *bcftools consensus* and the final *ITS1* sequence and associated BED file of mapped positions were extracted with *bedtools getfasta*. Consensus sequences needed to be corrected for missing coverage. When a nucleotide position is not covered by a mapped read, the called consensus nucleotide is assigned the reference value by *bcftools*. We used a custom R script to interrogate BED files to identify *ITS1* position with and without

supporting read coverage. Sites without coverage were assigned “?” for missing values. Samples with ≤5% missing nucleotides were retained for downstream analyses (Cluster 1A, n = 46; Cluster 1B, n = 26; and Cluster 2, n = 8).

Our *Acropora ITS1* sequences were also combined with *Acropora* ITS1 sequences from GenBank to visualise taxonomic relationships. These GenBank sequences included: *A. cervicornis*, AF239148 and AF239147; *A. cytherea*, AF538555 and AF538552; *A. longicyathus*, AF538580 and AF538579; *A. millepora*, KY408093 and KY408092; *A. spicifera*, AF538558 and AF538557; *A. tenuis*, AF538489 and AF538524; *A. valida*, KY408063 and KY408062; and as an outgroup, *Isopora palifera*, AY722744. ITS1 sequences were aligned using the ClustalW algorithm with R’s *msa* package (Bodenhofer, Bonatesta, Horejš-Kainrath, & Hochreiter, 2015). The *phangorn* (Schliep, 2011) function, *modelTest*, was used to determine whether the JC, F81, or K80 model best explained the multispecies alignment. The K80 model was the best and was used as the underlying evolutionary model to generate a genetic distance matrix, with the *dist.dna* function from the *ape* package (Paradis, Claude, & Strimmer, 2004). This genetic distance matrix was then used to build a neighbour joining tree with *ape*’s *nj* function. The final tree (Fig. S4) was visualised with the *ggtree* package (Yu, Smith, Zhu, Guan, & Lam, 2017).

Additionally, to better understand population-level diversity in our dataset, we visualised our data together with previously published *A. tenuis* populations from five inshore reefs (n = 148, (Cooke et al., 2020) using genotype likelihood estimation in *ANGSD* and PCA in *PCAngsd*. We also performed a clustering analysis in *STRUCTURE* (Pritchard, Stephens, & Donnelly, 2000) using previously published *A. tenuis* microsatellite data (Lukoschek, Riginos, & van Oppen, 2016) genotyped in the same individuals for which we have genomic data (Fig. S6).

Population genetic structure and demographic analysis among genetic clusters

To obtain cluster-specific diversity and neutrality statistics, we applied the Q15 quality filters and additionally retained only sites below a total read depth of three standard deviations above the cluster-specific mean. We used the folded SFS using realSFS and the -saf2theta option. We used the thetastat do_stat option to calculate statistics for each site and in overlapping 50kb windows (10kb step size) across the genome. We generated unfolded

2D-SFS for each comparison and calculated pairwise FST for each site and in overlapping 50kb windows (10kb step size).

Dadi optimization was based on the method outlined in Portik et al 2017. Briefly, this approach entails successive rounds of optimization, wherein different replicates (i.e., model with same parameters) are optimized each round with the best fitting parameters being used as starting parameters in the next round. For our analysis, we performed 5 rounds of optimization with 20 replicates being performed in the first four rounds and 50 replicates in the last round. Moreover, we increased the maximum iteration for each replicate to 20 for all rounds. The initial parameter values for each run were randomly selected from the range of values for each parameter (see Fig. S7 for models and parameters). For the extrapolation procedure of *dadi*, we used the linear extrapolation (i.e., make_extrap_func) rather than the logarithmic. To estimate confidence intervals for our parameter estimates, we performed a non-parametric bootstrapping. This procedure involved generating 150 bootstrap replicates for each of the seven pairwise comparisons. The bootstrap joint-SFS was generated using ANGSD v0.934. For each of the bootstrap replicates, we ran a similar *dadi* optimization as our empirical data set. However, we used the optimized parameter as a starting value, and we only performed one round of optimization composed of 50 replicates. The best fit parameters for each of the bootstrap replicate were then used to generate the distribution of the parameter. The estimated parameter values from our *dadi* analysis were converted to years and number of genomes following Cooke et. al. 2020. Specifically, we used a mutation rate of 1.86×10^{-8} per base per year – applied to 296, 667 positions we specified in estimating the SFS – in converting theta estimates to N_{ref}, and we used a generation time of 5 years to convert the time estimates to years.

Spatial distribution of cnidarian host genetic clusters relative to latitude and shelf position

Individual allele frequencies representing the response matrix **Y** were obtained from *PCAngsd*. Individual allele frequencies represent the likely dosage of the minor allele (0, a homozygote for the major allele; 0.5, a heterozygote; and 1, a homozygote for the minor allele). As a continuous measure, the individual allele frequency accounts for uncertainty in the true genotype. These individual allele frequencies were scaled such that each SNP locus had a mean of 0 and a variance of 1. Significance of the our RDA model, the axes, and terms were assessed using the *anova.cca* function in vegan (Oksanen et al., 2017). We considered statistical significance at an $\alpha < 0.05$. We tested the significance of terms using a Type I

sequential sums of squares by using the call by="terms" to test for the effect of latitude before testing the effect of shelf position.

Assaying symbiont diversity using ITS2 and plastid genomes

Mapping of coral short read sequences to the *Cladocopium* plastid sequences was performed with *bwa mem* and filtered for MAPQ ≥ 15 with *samtools*. Variants were called using *freebayes* using the "--pooled-continuous" option, to model unknown allele copies within samples, and the "--min-coverage" option, to require a minimum of 6 reads to support a variant call. Variant filtering was performed in R using an iterative pipeline. The R package *genomalicious* (Thia & Riginos, 2019) was used to import variants into R with the *vcf2DT* function. To characterise symbiont plasmid diversity, we first identified a set of plastid variants that were well covered in our set of representative coral samples. These plastid variants required a depth of ≥20 reads in >100 of our representative coral samples. We then removed samples with ≤30 loci with adequate read depth from each host genetic cluster, leaving n = 67, n = 37, and n = 4, respectively, for Clusters 1A, 1B and 2. From these remaining samples, we removed any loci with missing data, resulting in a final working set of 19 loci, 6 from contig 1 and 13 from contig 7. These working loci, derived from our best covered representative coral samples, were used to filter loci in our full variant dataset across all coral samples.

For the GLM of symbiont counts, we used the *lmer* function from the *lme4* package (Bates, Mächler, Bolker, & Walker, 2014) to fit the model: Counts was a continuous predictor, the number of reads mapped to a locus, log-transformed and scaled to a mean of 0 and a variance of 1. Latitude was fit as a continuous predictor, scaled to a mean of 0 and a variance of 1. Shore was fit as a categorical predictor for shore position: inshore, midshelf, and offshore. The effect of the host cluster was fit as a categorical predictor, the inferred genetic clusters: 1A, 1B, and 2. The effect of locus and sample were fit as random effects to allow a unique intercept for each plastid locus and each coral colony, respectively. Because there was autocorrelation among shore positions and host clusters with latitude (Figure 1 and 2), we tested statistical significance with a Type I sums of squares with the *anova* function. That is, the effect of read counts was tested before testing the effect of latitude, before testing the effect of shore position, before testing the effect of host cluster. We considered statistical significance at an $\alpha < 0.05$.

The partial RDA for symbionts was constructed using an allele count matrix with samples in rows, alleles in columns, and read counts in cells, with dimensions 223 × 72. In the allele count matrix, we scored alleles as dummy-coded factor levels: the *k* alleles at each locus were represented by *k* columns in the allele count matrix. This dummy-coded data structure is analogous to allelic dummy coding implemented in the population genetics algorithms in R's *adegenet* package (Jombart, 2008). We used the *rda* function from the *vegan* package to fit the partial RDA mode, where, the symbiont allele count matrix (**Y**) was scaled with a Hellinger transformation prior to analysis using *vegan*'s *decostand* function. Counts was a continuous predictor of total log-transformed read counts mapped to the symbiont plastid, scaled to mean of 0 and a variance of 1. The effect of read counts was used to condition the response matrix before testing the effects of latitude, shore and host cluster. Latitude was a continuous predictor, scaled to a mean of 0 and variance of 1. Shore and cluster were categorical variables: the inshore factor level was used as the reference for the shore effect, whilst the Cluster 1A factor level was the reference factor level for the host cluster effect. Significance of the RDA model, the axes, and terms was assessed using the *anova.cca* function. We considered statistical significance at an $\alpha < 0.05$. We tested the significance of terms using a Type I sequential sums of squares by using the call by="terms" to test for the effect of latitude, before testing the effect of shore, before testing the effect of host genetic cluster.

SUPPLEMENTARY LITERATURE CITED

Bates, D., Mächler, M., Bolker, B., & Walker, S. (2014). Fitting linear mixed-effects models using lme4. *arXiv.org*, 1303.3750.

Bodenhofer, U., Bonatesta, E., Horejš-Kainrath, C., & Hochreiter, S. (2015). msa: an R package for multiple sequence alignment. *Bioinformatics, 31*(24), 3997-3999.

Bongaerts, P., Riginos, C., Brunner, R., Englebert, N., Smith, S. R., & Hoegh-Guldberg, O. (2017). Deep reefs are not universal refuges: Reseeding potential varies among coral species. *Science Advances, 3*(2), e1602373. doi:papers3://publication/doi/10.1126/sciadv.1602373

Browning, S. R., & Browning, B. L. (2007). Rapid and accurate haplotype phasing and missing-data inference for whole-genome association studies by use of localized haplotype clustering. *The American Journal of Human Genetics, 81*(5), 1084-1097. doi:papers3://publication/doi/10.1086/521987

Bushnell, B. (2014). BBMap: A fast, accurate, splice-aware aligner. Report Number: LBNL-7065E. In: Lawrence Berkeley National Laboratory, Berkeley, CA.https://sourceforge.net/projects/bbmap/.

Cooke, I., Ying, H., Forêt, S., Bongaerts, P., Strugnell, J., Simakov, O., . . . Miller, D. J. (2020). Signatures of selection in the coral holobiont reveal complex adaptations to inshore environments driven by Holocene climate change. *Science Advances, 6*, eabc6318. doi:10.1101/2020.02.25.951905

Cowman, P. F., Quattrini, A. M., Bridge, T. C. L., Watkins-Colwell, G. J., Fadli, N., Grinblat, M., . . . Baird, A. H. (2020). An enhanced target-enrichment bait set for Hexacorallia provides phylogenomic resolution of the staghorn corals (Acroporidae) and close relatives. *Molecular Phylogenetics and Evolution, 153*, 106944. doi:10.1016/j.ympev.2020.106944

Garrison, E., & Marth, G. T. (2012). Haplotype-based variant detection from short-read sequencing. *arXiv.org, 1207.3907 [q-bio.GN]*.

Jombart, T. (2008). adegenet: a R package for the multivariate analysis of genetic markers. *Bioinformatics, 24*(11), 1403-1405. doi:papers3://publication/doi/10.1093/bioinformatics/btn129

Katoh, K., & Standley, D. M. (2013). MAFFT multiple sequence alignment software version 7: improvements in performance and usability. *Molecular Biology and Evolution, 30*(4), 772-780. doi:10.1093/molbev/mst010

Korneliussen, T. S., Albrechtsen, A., & Nielsen, R. (2014). ANGSD: analysis of next generation sequencing data. *Bmc Bioinformatics, 15*(1), 443. doi:papers3://publication/doi/10.1186/s12859-014-0356-4

Li, H. (2011). A statistical framework for SNP calling, mutation discovery, association mapping and population genetical parameter estimation from sequencing data. *Bioinformatics, 27*(21), 2987-2993. doi:10.1093/bioinformatics/btr509

Li, H. (2013). Aligning sequence reads, clone sequences and assembly contigs with BWA-MEM. *arXiv.org*, 1303.3997v1302

Li, H., & Durbin, R. (2009). Fast and accurate short read alignment with Burrows–Wheeler transform. *Bioinformatics, 25*(14), 1754-1760.

Li, H., Handsaker, B., Wysoker, A., Fennell, T., Ruan, J., Homer, N., . . . Durbin, R. (2009). The sequence alignment/map format and SAMtools. *Bioinformatics, 25*(16), 2078-2079.

Lukoschek, V., Riginos, C., & van Oppen, M. J. H. (2016). Congruent patterns of connectivity can inform management for broadcast spawning corals on the Great

Barrier Reef. *Molecular Ecology, 25*(13), 3065-3080. doi:papers3://publication/doi/10.1111/mec.13649

Manzello, D. P., Matz, M. V., Enochs, I. C., Valentino, L., Carlton, R. D., Kolodziej, G., . . . Jankulak, M. (2019). Role of host genetics and heat-tolerant algal symbionts in sustaining populations of the endangered coral *Orbicella faveolata* in the Florida Keys with ocean warming. *Global Change Biology, 25*(3), 1016-1031. doi:papers3://publication/doi/10.1111/gcb.14545

Meisner, J., & Albrechtsen, A. (2018). Inferring population structure and admixture proportions in low-depth NGS data. *Genetics, 210*(2), 719-731. doi:10.1534/genetics.118.301336

Minh, B. Q., Schmidt, H. A., Chernomor, O., Schrempf, D., Woodhams, M. D., von Haeseler, A., & Lanfear, R. (2020). IQ-TREE 2: new models and efficient methods for phylogenetic inference in the genomic era. *Mol Biol Evol, 37*(5), 1530-1534. doi:10.1093/molbev/msaa015

Morgulis, A., Gertz, E. M., Schäffer, A. A., & Agarwala, R. (2006). A fast and symmetric DUST implementation to mask low-complexity DNA sequences. *Journal of Computational Biology, 13*(5), 1028-1040.

Oksanen, J., Blanchet, F. G., Friendly, M., Kindt, R., Legendre, P., McGlinn, D., . . . Wagner, H. (2017). vegan: community ecology package. R package version 2.4-2. https://CRAN.R-project.org/package=vegan. doi:papers3://publication/uuid/4A89BCA6-F57F-4814-9242-8175EA259110

Paradis, E., Claude, J., & Strimmer, K. (2004). APE: analyses of phylogenetics and evolution in R language. *Bioinformatics, 20*(2), 289-290.

Pritchard, J. K., Stephens, M., & Donnelly, P. (2000). Inference of population structure using multilocus genotype data. *Genetics, 155*, 945-959.

Quinlan, A. R., & Hall, I. M. (2010). BEDTools: a flexible suite of utilities for comparing genomic features. *Bioinformatics, 26*(6), 841-842.

Riginos, C., Hock, K., Matias, A. M., Mumby, P. J., Oppen, M. J. H., & Lukoschek, V. (2019). Asymmetric dispersal is a critical element of concordance between biophysical dispersal models and spatial genetic structure in Great Barrier Reef corals. *Diversity and Distributions, 25*(11), 1684-1696. doi:papers3://publication/doi/10.1111/ddi.12969

Schliep, K. P. (2011). phangorn: phylogenetic analysis in R. *Bioinformatics, 27*(4), 592-593.

Skotte, L., Korneliussen, T. S., & Albrechtsen, A. (2013). Estimating individual admixture proportions from next generation sequencing data. *Genetics, 195*(3), 693-702.

Thia, J. A., & Riginos, C. (2019). genomalicious: serving up a smorgasbord of R functions for population genomic analyses. *bioRxiv*, 667337.

Wilson, K., Li, Y., Whan, V., Lehnert, S., Byrne, K., Moore, S., . . . Ballment, E. (2002). Genetic mapping of the black tiger shrimp *Penaeus monodon* with amplified fragment length polymorphism. *Aquaculture, 204*(3-4), 297-309.

Yu, G., Smith, D. K., Zhu, H., Guan, Y., & Lam, T. T. Y. (2017). ggtree: an R package for visualization and annotation of phylogenetic trees with their covariates and other associated data. *Methods in Ecology and Evolution, 8*(1), 28-36.

SUPPLEMENTARY FIGURE LEGENDS

Figure S1. Dendogram based on Identity-by-State (IBS) proportions for all 463 samples. Non-black colors correspond to similar MLGs. When all samples were included in estimating IBS, = the most distant clade (yellow clade) was identified as a single MLG.

Figure S2. The distribution of distances generated from IBS matrices calculated for samples belonging to the yellow clade (transparent distribution on the right) and all remaining samples (grey distribution at the center). The transparent distribution on the left indicates distances among yellow clade samples when IBS was calculated including all 463 samples, which resulted in all yellow clade samples being identified as a single MLG. The vertical red line is the mean IBS distance among our technical replicates, which served as a threshold for MLG identification.

Figure S3: Counts of individuals assigned to different clusters from Admixture with K=3. A threshold of 0.85 was used to assign individuals as representing parental types.

Figure S4: Phylogenetic trees based on coral host sequences. (a) Neighbour joining tree of coral host *ITS1* sequences. Node labels denote support for 250 bootstrap replicates. (b) Maximum likelihood IQ-Tree of cost using UCE and exon sequences. Node support was filtered for >0.8 support. For both trees, representative *A. tenuis* samples from our study ("Aten") were analysed in conjunction with other Acroporids and *Isopora palifera* as an outgroup. Colours represent genetic clusters (*NGSadmix* $q \geq 0.85$) versus GenBank references (see legend). Scale bar indicates the number of substitutions per unit length.

Figure S5: Microsatellite-based assignments using Structure for K=3 for individuals included in both this study and (Lukoschek et al., 2016; Riginos et al., 2019). Clusters are not clearly resolved as compared to results with genome wide SNPs (as shown by Fig. 1).

Figure S6: Distribution of F_{ST} estimated per 50 kb window across the genome. The heatmap shows the proportion of windows showing a certain level of F_{ST}. As expected, comparison between Cluster1a and Cluster1b resulted in low F_{ST} values with ~71% of the windows examined showing F_{ST} values between 0 - 0.02 (median F_{ST} = 0.075). In contrast, comparisons involving Cluster2 showed higher levels of F_{ST} with ~30% of the windows examined having F_{ST} > 0.18 (for both Cluster1a vs Cluster2 and Cluster1b vs Cluster2). For Cluster1a vs Cluster1b the mean F_{ST} value is 0.019 (median = 0.009; sd = 0.029); 0.160 for Cluster1a vs

Cluster2 (median = 0.080; sd = 0.190) and 0.162 for Cluster1b vs Cluster2 (median = 0.075; 0.203). F_{ST} estimates for 6, 235 windows were included in this plot.

Figure S7: Isolation by distance within each genetic cluster. We found a significant association between genetic differentiation and reef distance within Cluster 1 (Mantel test R=0.4373, p<0.01); however, the small number of reefs with greater than 3 individuals in cluster 1b (R=-0.1209, p=0.5) and cluster 2 (R=0.9991, p=0.17), did not yield significant correlations.

Figure S8: Estimates (a) Watterson's Theta, (b) nucleotide diversity, and (c) Tajima's D for the 5,194 genomic windows shared by the three genetic clusters distributed across 138 scaffolds of the reference genome.

Figure S9: (a) The four different demographic models that were fitted on the seven joint-SFS representing the different comparisons. Ne parameters ranged from 0.005 to 50 of the Nref; time parameters ranged from 0.001 to 15; and migration parameters ranged from 0 to 10. The log-likelihood of each model and its corresponding AIC (Akaike Information Criterion) for comparisons within Cluster1 are summarized in (b), while those comparisons involving Cluster 2 are summarized in (c). For (b) and (c), the grey bar for log-likelihood represents the 2.5% quantile up to the maximum log-likelihood (red line on top of the bar) obtained for the 50 replicates of the last round of the parameter optimization.

Figure S10. Parameter estimates for the four demographic models (a to d) fitted to the different joint-SFS (y-axis of each plot). (Row C is identical to Fig. 7 but is included here for completeness.) For each model, the leftmost panel includes a depiction of the model together with boxes indicating the clusters identity (color) and geographic location (labels: N – north; M – mid; S – south) of the individuals included in the analyses. The bars in the parameter estimate plots indicate the 2.5% - 97.5% quantile of the bootstrap estimates, while the red horizontal line shows the empirical estimate. These parameter estimates were converted from *dadi* parameters following Cooke et. al. 2020. Specifically, we used a mutation rate of 1.86 x 10^{-8} per base per year – applied to 296, 667 positions we specified in estimating the SFS – in converting theta estimates to N_{ref}; while a generation time of 5 years was used to convert time estimates to year.

SUPPLEMENTARY TABLES

Table S1. Sample information by colony

Dataset	Site Name	Sample owner [a]	Year collected	Numb sequenced	Numb retained post filtering [b]	Numb samples used for microsatellite analysis [c]
This study	Wallace Islet	AIMS	2011	27	23	23
	Great Detached Reef	AIMS	2009	36	32	28
	Long Sandy Reef	AIMS	2009	27	27	27
	Curd Reef	AIMS	2009	32	30	30
	Night Reef	AIMS	2011	31	31	31
	Sandbank No7	AIMS	2009	31	31	31
	Wilkie Reef	AIMS	2011	32	30	30
	Tydeman Reef	Lukoschek	2011	32	26	26
	No Name	Lukoschek	2011	29	22	22
	Lizard South Island	Lukoschek	2011	31	8	8
	Fitzroy Island	AIMS	2017	30	23	0
	Feather Reef	AIMS	2017	30	19	0
	Kelso Reef	AIMS	2011	32	28	28
	Orpheus Island	Lukoschek	2010	32	31	31

	Seagull Reef	AIMS	2013	20	11	11
	Bugatti Reef	AIMS	2009	30	22	22
	Daydream Reef	AIMS	2014	10	2	0
	Pine Reef	AIMS	2014	13	5	0
	Reef 20-344	AIMS	2013	23	16	16
	Reef 21-558	AIMS	2010	29	13	13
	Frigate Reef	AIMS	2010	32	9	9
	Great Keppel Island	AIMS	2010	11	7	7
	One Tree Island (Lagoon)	Lukoschek	2011	3	2	2
	Recreation Reef	?	?	31	0	0
	Wistari Reef (Heron Island)	?	?	32	0	0
	One Tree Island (Wall)	?	?	19	0	0
	Double Cone Reef	?	?	17	0	0
Total present study				*702*	*448*	*395*
Cooke et al. 2021	Fitzroy Island	AIMS	2015	NA	30	NA
	Dunk Island	AIMS	2015	NA	30	NA
	Pelorus Island	AIMS	2015	NA	30	NA
	Pandora Reef	AIMS	2015	NA	30	NA

	Magnetic Island	AIMS	2015	NA	28	NA
Total Cooke et al. 2021					*148*	*NA*

[a] AIMS: Australian Institute of Marine Science; private collections of Vimoksalehi Lukoschek.

[b] SNP datasets for cnidarian component of the colony.

[c] From Lukoschek et al. 2016

Table S2. Minimum quality criteria applied to choose a random sample of focal loci for target bait capture. Variants were extracted from the *A. tenuis* draft genome (aten_final_0.11.fasta) and population genomic dataset from Cooke et al (2021)

**Quality Criteria**
1. Minimum locus quality score of 30
2. Biallelic SNPs retained only
3. Minimum mean depth per individual 0.9 and max mean depth per individual of 5.4
4. Minimum genotype quality 20 (applied to genotypes)
5. Maximum missing genotypes at a locus 50% (applied after step 4)
6. Allele balance probability (calculated by freebayes [a]) < 20
7. SNPs in low complexity regions (identified by mdust [b]) removed
8. SNPs within 5bp (either direction) of an indel removed
9. Remove SNPs with a minor allele count less than 3

[a] (Garrison & Marth, 2012)

[b] (Morgulis, Gertz, Schäffer, & Agarwala, 2006)

Table S3. The mean number of alleles per symbiont plastid locus for coral colonies belonging to different host genetic clusters.

**Cluster**	**1_4211**	**1_4214**	**1_4221**	**1_4230**	**1_4243**	**1_4244**	**7_266**	**7_271**	**7_277**	**7_283**
1A	1.80	1.92	1.22	4.22	1.14	1.09	1.58	1.97	1.75	1.72
1B	1.86	2.17	1.00	4.86	1.02	1.19	1.60	1.88	1.93	1.83
2	1.80	2.00	1.00	4.80	1.00	1.00	1.80	1.80	1.80	1.80

**Cluster**	**7_288**	**7_293**	**7_294**	**7_303**	**7_309**	**7_317**	**7_322**	**7_432**	**7_436**
1A	1.14	1.93	1.26	1.19	1.91	1.07	1.14	1.13	2.93
1B	1.05	2.00	1.10	1.10	2.00	1.14	1.02	1.12	2.71
2	1.20	2.00	1.00	1.00	2.00	1.00	1.20	1.00	2.20

Table S4. The mean number of alleles per symbiont plastid locus for coral colonies at different shore positions.

**Shore**	**1_4211**	**1_4214**	**1_4221**	**1_4230**	**1_4243**	**1_4244**	**7_266**	**7_271**	**7_277**	**7_283**
Inshore	1.71	1.77	1.31	3.59	1.20	1.04	1.45	2.00	1.63	1.61
Midshelf	1.86	1.71	1.29	4.43	1.00	1.14	2.00	2.00	1.86	1.86
Offshore	1.91	2.19	1.03	5.12	1.03	1.17	1.70	1.90	1.93	1.86

**Shore**	**7_288**	**7_293**	**7_294**	**7_303**	**7_309**	**7_317**	**7_322**	**7_432**	**7_436**
Inshore	1.21	1.89	1.37	1.23	1.86	1.06	1.21	1.15	3.28
Midshelf	1.00	1.86	1.14	1.00	1.86	1.14	1.14	1.29	2.57
Offshore	1.05	2.00	1.07	1.12	2.00	1.10	1.02	1.09	2.48
